# d‐Lactic acid secreted by *Chlorella fusca* primes pattern‐triggered immunity against *Pseudomonas syringae* in Arabidopsis

**DOI:** 10.1111/tpj.14661

**Published:** 2020-01-27

**Authors:** Sang‐Moo Lee, Seon‐Kyu Kim, Nakyeong Lee, Chi‐Yong Ahn, Choong‐Min Ryu

**Affiliations:** ^1^ Molecular Phytobacteriology Laboratory Korea Research Institute of Bioscience and Biotechnology (KRIBB) Daejeon 34141 South Korea; ^2^ Department of Biosystems and Bioengineering KRIBB School of Biotechnology University of Science and Technology Daejeon 34113 South Korea; ^3^ Personalized Genomic Medicine Research Center KRIBB Daejeon 34141 South Korea; ^4^ Cell Factory Research Center KRIBB Daejeon 34141 South Korea; ^5^ Department of Environmental Biotechnology KRIBB School of Biotechnology University of Science and Technology Daejeon 34113 South Korea

**Keywords:** biological control, *Chlorella fusca*, defence priming, d‐lactic acid, induced resistance, PAMP‐triggered immunity, pathogen‐associated molecular pattern

## Abstract

Biological control agents including microbes and their products have been studied as sustainable crop protection strategies. Although aquatic microalgae have been recently introduced as a biological control agent, the underlying molecular mechanisms are largely unknown. The aim of the present study was to investigate the molecular mechanisms underlying biological control by microalga *Chlorella fusca*. Foliar application of *C. fusca* elicits induced resistance in *Arabidopsis thaliana* against *Pseudomonas syringae* pv. tomato DC3000 that activates plant immunity rather than direct antagonism. To understand the basis of *C. fusca*‐triggered induced resistance at the transcriptional level, we conducted RNA sequencing (RNA‐seq) analysis. RNA‐seq data showed that, upon pathogen inoculation, *C. fusca* treatment primed the expression of *cysteine‐rich receptor‐like kinases*, *WRKY* transcription factor genes, and salicylic acid and jasmonic acid signalling‐related genes. Intriguingly, the application of *C. fusca* primed pathogen‐associated molecular pattern ‐triggered immunity, characterized by reactive oxygen species burst and callose deposition, upon flagellin 22 treatment. The attempts to find *C. fusca* determinants allowed us to identify d‐lactic acid secreted in the supernatant of *C. fusca* as a defence priming agent. This is the first report of the mechanism of innate immune activation by aquatic microalga *Chlorella* in higher plants.

## Significance Statement

Aquatic microalgae have been recently used as bioprotectants and biostimulants in crop plants; however, the underlying molecular mechanism remains unclear. Here, we demonstrate for the first time that the priming of PAMP‐triggered immunity induced by the aquatic microalgae *Chlorella fusca* against *Pseudomonas syringae* pv. tomato DC3000 in land plant *Arabidopsis*.

## Introduction

In the 20th century, chemical control methods were utilized mainly to manage plant diseases in modern agriculture due to their high efficacy and consistent effectiveness (Agrios, 2005). However, long‐term use of synthetic agrochemicals has negatively impacted human health, causes environmental pollution, and generated chemical‐resistant pathogens and herbivores (Bottrell and Smith, 1982). To overcome the limitations of agrochemicals, biological control has emerged as an alternative method of crop protection (Cook and Baker, 1983; Pal and McSpandden Gardener, 2006). Biological control is defined as ‘the reduction of the amount of inoculum density or disease‐producing activity of a plant pathogen accomplished by or through one or more organisms’ (Cook and Baker, 1983). To develop biological methods of crop protection, diverse microorganisms including beneficial bacteria, fungi, and yeast have been studied for determining their potential as biological control agents (Cook and Baker, 1983; Pal and McSpandden Gardener, 2006). Biological control agents suppress the growth of phytopathogens by producing antimicrobial compounds, inducing competition for limiting resources or physical space, and utilizing hyperparasitism (Cook and Baker, 1983; Pal and McSpandden Gardener, 2006). Additionally, biological control agents protected the host plant against phytopathogens by activating a type of plant immunity referred to as induced resistance (Kloepper *et al.*, 1992; Pieterse *et al.*, 1996; Fravel *et al.*, 2003; Segarra *et al.*, 2009).

Induced resistance is an enhanced state of plant innate immunity attained upon the application of an inducing agent against a broad spectrum of phytopathogens (Kloepper *et al.*, 1992; Pieterse *et al.*, 1996; Ryals *et al.*, 1996; Durrant and Dong, 2004). Application of inducing agents to a plant region spatially separated from the pathogen activates induced resistance systemically throughout the plant (Kloepper *et al.*, 1992; Pieterse *et al.*, 1996). Defence priming is an important feature of induced resistance, which increases the strength and speed of the immune response against future pathogen infection (Conrath *et al.*, 2002; Conrath *et al.*, 2006; Conrath *et al.*, 2015; Mauch‐Mani *et al.*, 2017). Induced resistance is generally trigged by certain agents such as avirulent pathogens, plant growth‐promoting rhizobacteria, and specific chemicals (Ross, 1961; Kuć, 1982; Kloepper *et al.*, 1992; Zimmerli *et al.*, 2000). In many cases, bacterial cell‐surface compounds and secreted compounds such as lipopolysaccharides, siderophores, 2,4‐diacetylphloroglucinol, flagellin, and volatile organic compounds act as induced resistance‐eliciting agents (van Peer and Schippers, 1992; Maurhofer *et al.*, 1994; Iavicoli *et al.*, 2003; Ryu *et al.*, 2004; Meziane *et al.*, 2005). However, the eukaryotic microalga itself or its derived agent that elicits induced resistance remain largely unknown.

*Chlorella* species are eukaryotic photosynthetic microorganisms found in freshwater, seawater, air, and soil (Liu and Chen, 2016). Because *Chlorella* species are rich in amino acids, carbohydrates, lipids, vitamins, and minerals, they are used as dietary supplements, feed additives, and biofuels (Liu and Chen, 2016). *Chlorella* culture or extract has been recently used a biological control agent against pathogenic nematodes and fungi in various agricultural crops. Irrigation of grapevine with the dried extract of *C. vulgaris* reduced the population of the pathogenic nematode *Xiphinema index* in the roots of grapevines (Bileva, 2013). Additionally, foliar spray of *C. vulgaris* decreased the decay rate of strawberry fruits and leafy vegetables, including lettuce, beet, and kale (Kim *et al.*, 2014). Foliar application of *C. fusca* reduced the incidence of disease caused by the fungal pathogen *Colletotrichum orbiculare* on cucumber leaves (Lee *et al.*, 2016; Kim *et al.*, 2018). However, the mechanism of *Chlorella*‐mediated biological control, such as induced resistance of host plant or direct antagonism, against pathogenic bacteria is largely unknown. In addition, despite the success of *Chlorella* as a biological control agent in agricultural crops, *Chlorella*‐derived determinants involved in the interaction between *Chlorella* and the host plant have not been reported previously.

Here, to elucidate the molecular mechanisms underlying biological control using microalgae, we used the model pathosystems, *Arabidopsis thaliana* and *Pseudomonas syringae* pv. tomato DC3000 (*Pto* DC3000) and, based on previous studies, we selected *C. fusca* as the model microalga species. We found that foliar application of *C. fusca* elicited induced resistance in Arabidopsis against *Pto* DC3000 through priming the expression of defence‐related genes involved in receptor kinases, transcription factors, and hormone signalling. Interestingly, foliar application of *C. fusca* primed pathogen‐associated molecular pattern (PAMP)‐triggered immunity (PTI), characterized by reactive oxygen species (ROS) burst and callose deposition, after PAMP treatment in Arabidopsis. Additionally, d‐lactic acid was identified as a *C. fusca*‐derived determinant, which primed induced resistance against *Pto* DC3000 and PAMP‐triggered immunity (PTI) response upon PAMP treatment in Arabidopsis. This report is the first to describe the molecular mechanism of plant innate immunity triggered by the interaction between aquatic microalga *Chlorella* and the land plant Arabidopsis through the secretion of a *Chlorella*‐derived isomeric molecule.

## Results

### Plant protection against *Pto* DC3000 by spray application of *C. fusca*

**Figure 1:**
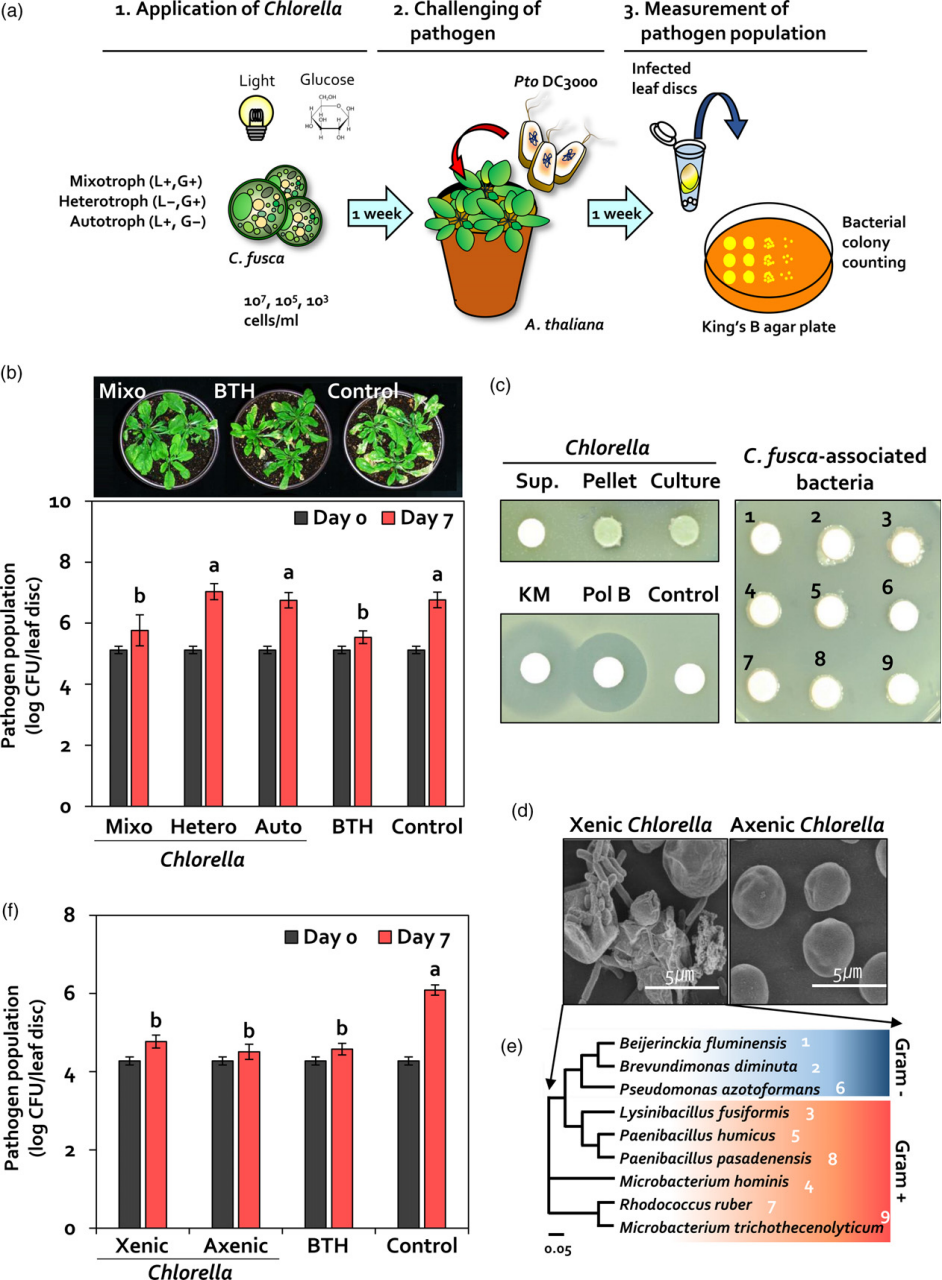
*Chlorella fusca* elicits induced resistance in Arabidopsis. (a) Leaves of 3‐week‐old Arabidopsis plants were sprayed with 10^7^, 10^5^, or 10^3^ cells ml^−1^ of *C. fusca* culture. *Pto* DC3000 (OD_600_ = 1) was sprayed on Arabidopsis leaves at 7 days after *C. fusca* treatment until run‐off. At 0 and 7 days post‐inoculation (dpi), the population density of *Pto* DC3000 in infected leaves was calculated on a King’s B agar plate by counting colony forming units (cfu). (b) Bacterial cell count in *C. fusca*‐treated Arabidopsis leaves at 0 and 7 dpi. Mixotroph (Mixo), *C. fusca* grown under mixotrophic conditions; heterotroph (Hetero), *C. fusca* grown under heterotrophic condition; autotroph (Auto), *C. fusca* grown under autotrophic condition; BTH, 0.33 mM BTH; Control, BG11 broth medium. (c, d) Identification of *C. fusca*‐associated bacteria in a xenic mixotrophic *C. fusca* culture. Nine bacterial species were isolated from xenic *C. fusca* culture. To validate the effect of *C. fusca*‐associated bacteria species on plant immunity, axenic cultivation of *C. fusca* was conducted in BG11 medium containing 5 µg/ml vancomycin and 2 µg/ml polymyxin B. (e) Bacterial cell count in xenic and axenic *C. fusca*‐treated Arabidopsis leaves at 0 and 7 dpi. Xenic, mixotrophic *C. fusca* grown under xenic conditions; Axenic, mixotrophic *C. fusca* grown under axenic conditions; BTH, 0.33 mM BTH; Control, BG11 broth medium. (f) Co‐cultivation of *C. fusca* and *Pto* DC3000. Photograph was taken at 2 dpi of 1 × 10^7^ cells ml^−1^ *C. fusca* culture, 50 μg ml^−1^ kanamycin, 2 μg ml^−1^ polymyxin B, nine symbiotic bacteria (OD_600_ = 1), and BG11 medium on a lawn of *Pto* DC3000 cell suspension. Sup., supernatant of *C. fusca*; Pellet, cell pellet of *C. fusca*; Culture, *C. fusca* culture; 1, *Beijerinckia fluminensis*; 2, *Brevundimonas diminuta*; 6, *Pseudomonas azotoformans*; 3, *Lysinibacillus fusiformis*; 5, *Paenibacillus humicus*; 8, *Paenibacillus pasadenensis*; 4, *Microbacterium hominis*; 7, *Rhodococcus ruber*; and 9, *Microbacterium trichothecenolyticum*. Data represent mean ± standard error of the mean (SEM; *n* = 12 replications per treatment). Different letters indicate significant differences between treatments (*P* < 0.05; least significant difference, LSD).

To optimize conditions for the use of *C. fusca* as a biological control agent against *Pto* DC3000, *C. fusca* was cultivated under mixotrophic (light and glucose), heterotrophic (glucose without light), and autotrophic (light without glucose) conditions. *C. fusca* culture was sprayed on *Arabidopsis thaliana* ecotype Columbia (Col‐0) seedlings at a concentration of 10^7^, 10^5^, or 10^3^ cells ml^−1^ (Figure 1a). In leaves treated with 10^7^ cells ml^−1^ *C. fusca* culture grown under mixotrophic, heterotrophic, and autotrophic conditions, the population density of *Pto* DC3000 was 5.8 × 10^5^, 1.1 × 10^7^, and 5.6 × 10^6^ colony forming units (cfu) per leaf disc (diameter = 0.8 cm) respectively at 7 days post‐inoculation (dpi) of *Pto* DC3000 (Figure 1b). By contrast, the population density of *Pto* DC3000 was 3.5 × 10^5^ and 5.8 × 10^6^ cfu per leaf disc in Arabidopsis leaves pre‐treated with 0.33 mM benzothiadiazole (BTH) (positive control) and BG11 medium (negative control), respectively (Figure 1b). No significant difference was observed in pathogen population density between Arabidopsis leaves treated with *C. fusca* (at two concentrations: 10^5^ and 10^3^ cells ml^−1^) and those treated with BG11 medium (Figure S1). Collectively, these results indicated that mixotrophic *C. fusca* culture containing 10^7^ cells ml^−1^ was the most effective against *Pto* DC3000.

### Validation of induced resistance

To investigate the mechanism of biological control by *C. fusca*, we considered four mechanisms of biological control: (1) production of antimicrobial compounds, (2) competition for limiting resources or physical space, (3) hyperparasitism, and (4) activation of induced resistance in the host plant (Cook and Baker, 1983; Pal and McSpandden Gardener, 2006). As hyperparsitism is mostly used by fungi, it was excluded as a possible mechanism of biological control used by *C. fusca*. Moreover, because *C. fusca* is an aquatic organism, competition between *C. fusca* and *Pto* DC3000 in Arabidopsis leaves was unlikely. Additionally, as *C. fusca* and *Pto* DC3000 were not spatially separated in our system, we hypothesized that *C. fusca* produces an antibacterial compound against *Pto* DC3000. To test this hypothesis, *C. fusca* was co‐cultivated with *Pto* DC3000 on TSA agar medium (Figure 1c). Two antibiotics kanamycin and polymyxin B that are effective against Gram‐negative bacteria inhibited the growth of *Pto* DC3000 (Figure 1c, bottom left panel). However, no inhibition zone of *Pto* DC3000 was observed around the *C. fusca* supernatant‐, pellet‐, and culuture‐inoculated disc on TSA agar medium (Figure 1c, top left panel). These results indicated that *C. fusca* exhibited no antibacterial capacity against *Pto* DC3000 *in vitro*.

In nature, symbiotic bacteria tightly interact with *Chlorella* species, affecting the growth and physiology of *Chlorella* (Cho *et al.*, 2015). We observed the presence of bacteria on *C. fusca*‐inoculated TSA agar medium (Figure 1c, top left panel) and *C. fusca* culture solution (Figure 1d). To determine whether *Chlorella*‐associated bacteria exhibited antibacterial activity, nine bacterial isolates were isolated from *C. fusca* culture (Figure 1d,e)*.* These nine isolates were classified as *Microbacterium trichotecenolyticum*, *Brevundimonas diminuta, Rhodococcus ruber, Microbacterium hominis*, *Beijerinckia fluminensis, Pseudomonas azotoformans, Lysinibacillus fusiformis, Paenibacillus humicus,* and *Paenibacillus pasadenensis.* Among these isolates*, M. trichotecenolyticum, B. diminuta,* and *Rhodococcus* spp. have been previously reported as phycospheric bacterial symbionts of microalgae (Watanabe *et al.*, 2005; Lian *et al.*, 2018; Pastore and Sforza, 2018). To investigate the antibacterial capacity of *C. fusca*‐associated bacteria, each of the nine bacterial isolates was co‐cultivated with *Pto* DC3000 (Figure 1c, right panel). No inhibition zone of *Pto* DC3000 was observed around discs inoculated with any of these nine bacterial isolates. This result indicates that *C. fusca*‐associated bacteria do not exhibit antagonistic activity against *Pto* DC3000.

Based on the results of antagonism, we hypothesized that *C. fusca* employs the activation of induced resistance in Arabidopsis as a potential mechanism to protect Arabidopsis against *Pto* DC3000. However, the possiblity that *C. fusca*‐associated bacteria, but not *C. fusca,* affected induced resistance was not excluded. To assess induced resistance triggered by *C. fusca*‐associated bacteria, we prepared axenic and xenic *C. fusca* cultures under mixotrophic conditions (Figure 1d,f). At 7 dpi, the population density of *Pto* DC3000 in Arabidopsis leaves pre‐treated with xenic and axenic *C. fusca* cultures was 5.9 × 10^4^ and 3.2 × 10^4^ cfu per leaf disc, respectively (Figure 1f). By contrast, the population density of *Pto* DC3000 in BG11 medium‐treated Arabidopsis leaves was 1.2 × 10^6^ cfu per leaf disc at 7 dpi (Figure 1f). These results suggest that *C. fusca‐*associated phycosphere bacteria do not contribute to the direct reduction in *Pto* DC3000 growth and induced resistance. Thus, in addition to *Chlorella*‐associated bacteria, *C. fusca* alone is able to trigger induced resistance in Arabidopsis against *Pto* DC3000.

### Transcriptome analysis of *C. fusca*‐triggered induced resistance

**Figure 2:**
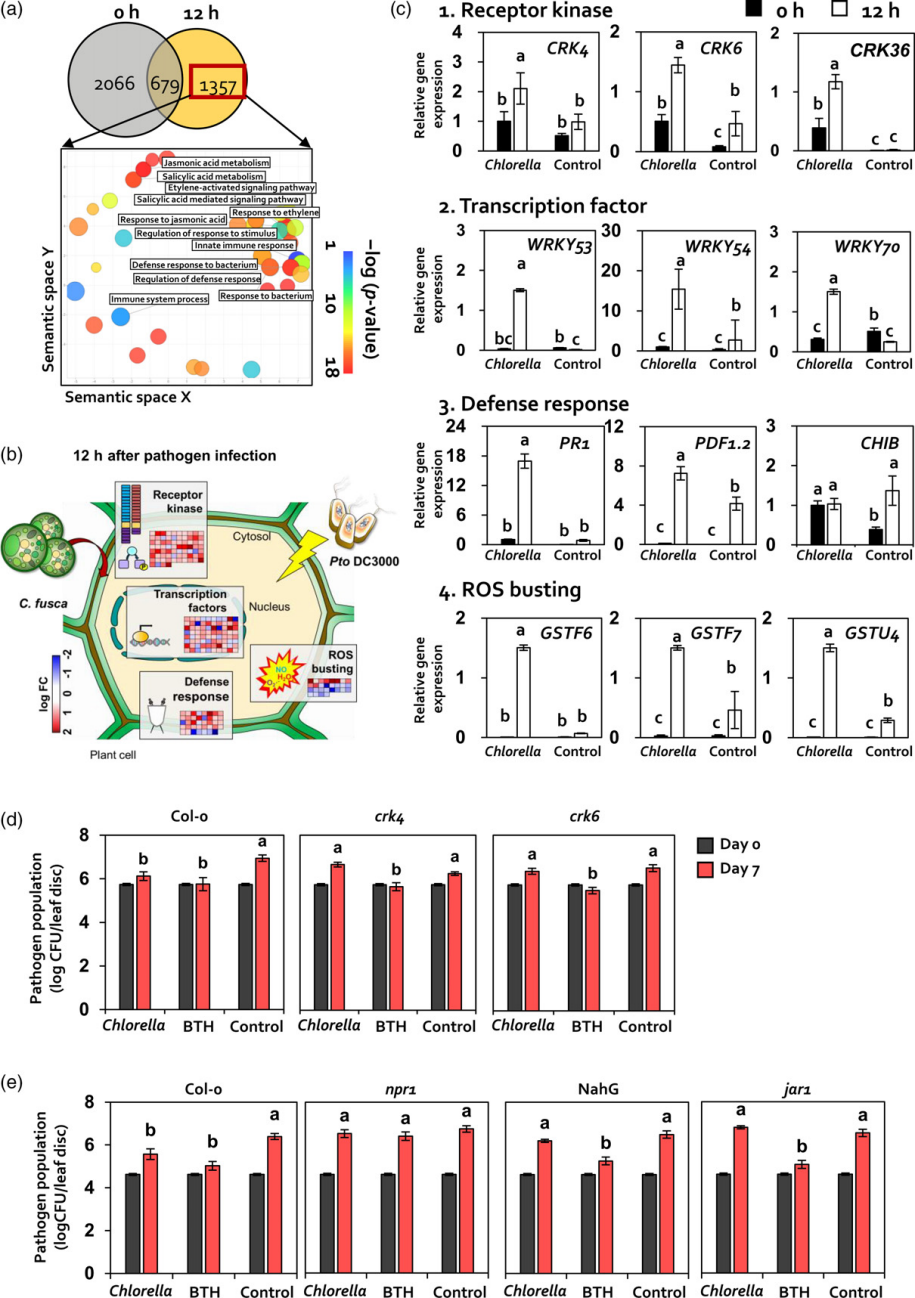
*C. fusca* primes the expression of defence signalling in Arabidopsis. (a) Venn diagram (upper panel) and gene ontology (GO) enrichment analysis (lower panel) of differentially expressed genes (DEGs) in *C. fusca*‐treated Arabidopsis at 0 and 12 hpi. GO enrichment analysis was performed using DAVID, and data were visualized using ReviGO software. The threshold of significance was −log (*P* < 0.05). (b) Functional classification of DEGs in *C. fusca*‐treated versus untreated Arabidopsis leaves at 12 hpi. *C. fusca*‐treated Arabidopsis leaves showed higher expression of genes associated with ‘Receptor kinase’, ‘Transcription factors’, ‘Defence response’, and ‘ROS busting’ than untreated leaves. MapMan software was used to visualize changes in the transcript abundance of DEGs (with at least two‐fold difference in expression) involved in stress response. Red squares indicate upregulated genes, and blue squares indicate downregulated genes. (c–e) Validation of transcriptomic analysis via qRT‐PCR and Arabidopsis mutant lines. (c) Expression pattern of DEGs in *C. fusca*‐treated and *C. fusca*‐untreated Arabidopsis leaves at 0 and 12 hpi. Four functional groups of DEGs are associated with ‘Receptor kinase’, ‘Transcription factor’, ‘Defence response’, and ‘ROS bursting’. *Chlorella*, *C. fusca*‐treated Arabidopsis; Control, *C. fusca*‐untreated Arabidopsis. Different letters indicate significant differences between treatments (*P* < 0.05; LSD). Data represent mean ± SEM. *Chlorella*, *C. fusca*‐treated Arabidopsis; Control, *C. fusca*‐untreated Arabidopsis; 0 h, 0 h post‐inoculation with *Pto* DC3000; 12 h, 12 h post‐inoculation of *Pto* DC3000. (d, e) Bacterial cell count in *C. fusca‐*treated *crk4* and *crk6* mutants (d), and *npr1* and *jar1‐1* mutants and NahG overexpression line (e). Data represent mean ± SEM (*n* = 12 replications per treatment). Different letters indicate significant differences between treatments (*P* < 0.05; LSD).

To examine the activation of *C. fusca*‐triggered induced resistance in Arabidopsis, we conducted RNA‐seq analysis of Arabidopsis leaves treated with and without *C. fusca* at 0 and 12 h post‐inoculation (hpi) with *Pto* DC3000 (Figure 2). We identified genes with >2‐fold differences in expression between *C. fusca*‐treated and *C. fusca* ‐untreated Arabidopsis leaves at 0 and 12 hpi with *Pto* DC3000. In total, 2066 and 1357 differentially expressed genes (DEGs) were identified in *C. fusca*‐treated Arabidopsis leaves at 0 and 12 hpi, respectively (Figures 2a and S2). Additionally, 679 genes were common to both time points (0 and 12 hpi) with *Pto* DC3000.

To analyze the functional categorization of DEGs associated with induced resistance triggered by *C. fusca*, we conducted gene ontology (GO) analysis of DEGs identified at 12 hpi with *Pto* DC3000 using the DAVID database and ReviGO software (Figure 2a). DAVID database analysis showed that numerous DEGs were associated with ‘immune system process’, ‘innate immune response’, and ‘response to bacterium’ under the biological process category at 12 hpi with *Pto* DC3000 (Figure S3). In addition, ReviGO analysis showed significant enrichment of DEGs involved in salicylic acid (SA), jasmonic acid (JA), and ethylene (ET) signalling under the biological process category at 12 hpi with *Pto* DC3000 (Figure 2a,c).

To determine the defence mechanisms induced by *C. fusca*, MapMan tool analysis was conducted on DEGs identified in *C. fusca*‐treated Arabidopsis sample at 12 hpi (Figure 2b). The results showed that *C. fusca* triggered a series of defence mechanisms at 12 hpi by activating genes belonging to four functional groups: (1) perception (i.e., recognition of external signals by receptor kinases), (2) transcription factors, (3) defence signalling, and (4) ROS bursting. In total, 74 DEGs exhibiting receptor kinase activity were identified, including the *cysteine‐rich receptor‐like kinase* (*CRK*) family genes *CRK4*, *CRK6*, and *CRK36*, most of which were upregulated by *C. fusca* at 12 hpi with *Pto* DC3000 (Figure 2b). Additionally, 80 DEGs encoding transcription factors were identified at 12 hpi; these included transcription factor‐encoding *WRKY* group III genes (e.g. *WRKY53*, *WRKY54*, and *WRKY70*) involved in plant defence. Among genes involved in multiple defence signalling pathways, *C. fusca* application highly upregulated 39 DEGs involved in SA and JA signalling, including *pathogenesis‐related 1* (*PR1*) and *plant defensin 1.2 (PDF1.2*), respectively, and 28 DEGs involved in ROS burst, including *glutathione S‐transferase* genes *GSTF6*, *GSTF7*, and *GSTU4* at 12 hpi with *Pto* DC3000 (Figure 2b).

To validate the RNA‐seq data, the expression pattern of genes in four functional groups was investigated using quantitative reverse transcription PCR (qRT‐PCR). Except for expression of the *Basic Chitinase* (*CHIB*) gene, expression levels of genes involved in defence signalling, including *CRKs* (*CRK4*, *CRK6*, and *CRK36*) and *WRKY* group III genes (*WRKY53*, *WRKY54*, and *WRKY70*), as well as those involved in hormone signalling (*PR1* and *PDF1.2*) and ROS burst (*GSTF6*, *GSTF7*, and *GSTU4*) were significantly upregulated by >2‐fold in *C. fusca*‐treated Arabidopsis leaves compared with control leaves at 12 hpi with *Pto* DC3000 (Figure 2c). Furthermore, *C. fusca*‐treated Arabidopsis leaves showed defence priming of the expression of nine defence‐related genes (*CRK4*, *WRKY53*, *WRKY54*, *WRKY70*, *PR1*, *PDF1.2*, *GSTF6*, *GSTF7*, and *GSTU4*), which did not show a difference in transcript levels at 0 hpi but were significantly upregulated at 12 hpi with *Pto* DC3000 (Figure 2c). In addition, no significant differences in pathogen population density were observed between *C. fusca*‐treated and BG11 medium‐treated leaves of Arabidopsis mutants *crk4*, *crk6*, *npr1* (SA signalling mutant), and *jar1* (JA signalling mutant), and *NahG* overexpression line (Figure 2d,e). Collectively, the results of our transcriptome and mutant analyses demonstrate that application of *C. fusca* elicits the priming of defence‐related gene expression, which triggers induced resistance against *Pto* DC3000 in Arabidopsis.

### *C. fusca*‐triggered PTI priming

**Figure 3:**
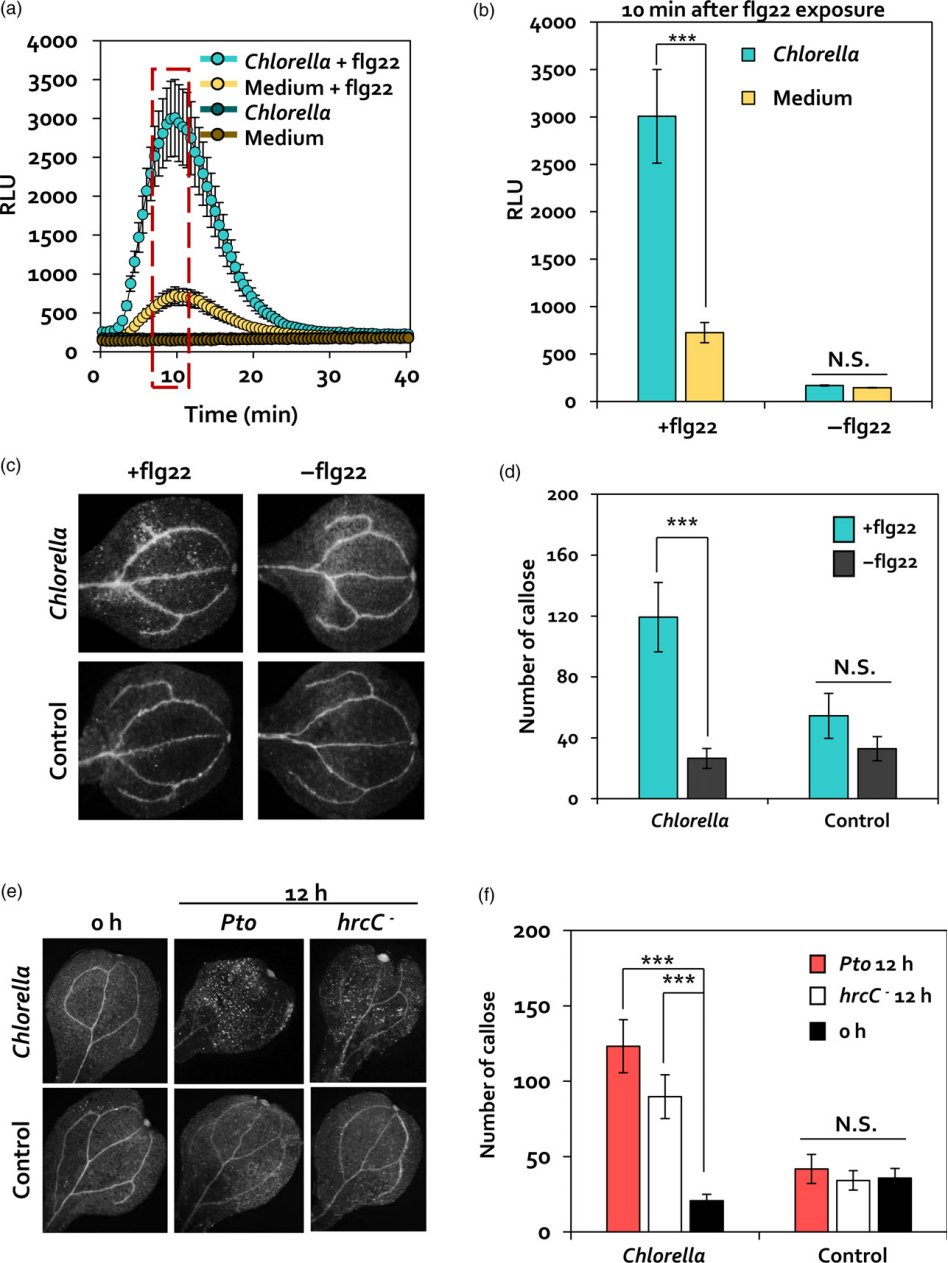
Elicitation of PTI priming by *C. fusca.* (a, b) Measurement of ROS burst in leaves of 4‐week‐old *C. fusca*‐treated Arabidopsis plants upon flg22 (100 nm) treatment. ROS production, measured as relative light units (RLUs), was evaluated for 40 min after flg22 treatment. Maximum RLU signal was detected at 10 min after flg22 exposure. Data represent the mean ± SE of three independent biological replicates, each consisting of six leaf discs (*n* = 18). Asterisks indicate significant differences (*P < 0.05, **P < 0.001, ***P < 0.0001). *Chlorella* + flg22, Arabidopsis leaves pre‐treated with *C. fusca* and subsequently treated with 100 nm flg22; Medium + flg22, Arabidopsis leaves pre‐treated with BG11 medium and subsequently treated with 100 nm flg22; *Chlorella*, Arabidopsis leaves pre‐treated with *C. fusca*; Medium, Arabidopsis leaves pre‐treated with BG11 medium (c, d) Callose deposition in *C. fusca*‐treated (*Chlorella*) or BG11 medium‐treated (Control) Arabidopsis seedlings upon 100 nm flg22 treatment. Cotyledons were collected at 1 h after flg22 exposure for aniline blue staining. +flg22, 1 h after flg22 treatment; −flg22, before flg22 treatment. (e, f) Callose deposition in *Chlorella*‐treated (*Chlorella*) or BG11 medium‐treated (Control) Arabidopsis seedlings upon *Pto* DC3000 (OD_600_ = 1) or *Pto* DC3000 *hrcC* ^−^ (OD_600_ = 1) inoculation. Cotyledons were collected at 12 hpi for aniline blue staining. Wild‐type 12 h, 12 h after *Pto* DC3000 inoculation; *hrcC* ^−^ 12 h, 12 h after *Pto* DC3000 *hrcC* ^−^ inoculation; 0 h, before pathogen inoculation. Data represent mean and SE of three independent biological replicates (*n* = 15 replications per treatment). Asterisks indicate significant differences (*P < 0.05, **P < 0.01, ***P < 0.001).

Because *C. fusca* induced the expression of *CRK* genes that are known to positively affect the PTI response characterized by ROS burst and callose deposition (Yeh *et al.*, 2015; Lee *et al.*, 2017), we examined whether *C. fusca* affects flagellin 22 (flg22)‐triggered PTI in Arabidopsis. ROS burst and callose deposition were detected in *C. fusca*‐treated Arabidopsis leaves using a luminol assay and aniline blue staining upon exposure to flg22 (Figure 3a,b). Arabidopsis leaves treated with *C. fusca* (*Chlorella*) and BG11 medium (Medium) showed no relative light unit (RLU) signal, an indicator of ROS (hydrogen peroxide and superoxide anion) production, during the detection period (Figure 3a). At 10 min after flg22 exposure, the RLU signal of leaves treated with *C. fusca* and subsequently treated with flg22 (*Chlorella* + flg22) was 4.14‐fold higher than that of leaves treated with BG11 medium and subsequently treated with flg22 (Medium + flg22) (Figure 3a,b). Consistent with the ROS burst results, no difference was detected in callose deposition between leaves treated with *C. fusca* (*Chlorella,* −flg22) and BG11 medium (Control, −flg22); however, upon flg22 treatment, callose deposition was 4.49‐fold higher in leaves treated with *C. fusca* and subsequently treated with flg22 (*Chlorella,* +flg22) than that of leaves treated with BG11 medium and subsequently treated with flg22 (Control, +flg22) (Figure 3c,d). These results suggested that *C. fusca* primes PTI responses in Arabidopsis.

To validate these results using live *Pto* DC3000 cells, Arabidopsis leaves were treated with a cell suspension of wild‐type *Pto* DC3000 rather than with flg22 (Figure 3e,f). Callose deposition was no different in *C. fusca*‐treated samples (*Chlorella*, 0 h) and the BG11 medium control (Control, 0 h). However, callose deposition in *C. fusca*‐treated leaves at 12 h after treatment with pathogen cell suspension (*Chlorella, Pto* 12 h) was 2.94‐fold higher than that in the BG11 medium control (Control*, Pto* 12 h) (Figure 3e,f). To eliminate potential side effects of *Pto* DC3000 effector proteins on the priming of callose deposition, Arabidopsis leaves were treated with live *Pto* DC3000 *hrcC* ^−^ mutant cells (Figure 3e,f); this mutant is defective in the secretion of type III effectors and therefore is unable to repress the PTI response. At 12 h after exposure to the *hrcC* ^−^ mutant cell suspension, callose deposition was 2.63‐fold higher in *C. fusca*‐treated leaves (*Chlorella*, *hrcC* ^−^ 12 h) than in BG11‐treated leaves (Control, *hrcC* ^−^ 12 h) (Figure 3e,f). These results indicate that *C. fusca* primes the PTI response through independent of effector proteins. Taken together, our results indicated that foliar application of *C. fusca* primes PTI in Arabidopsis in response to PAMP molecules.

### Identification of *C. fusca* determinants conferring induced resistance

**Figure 4:**
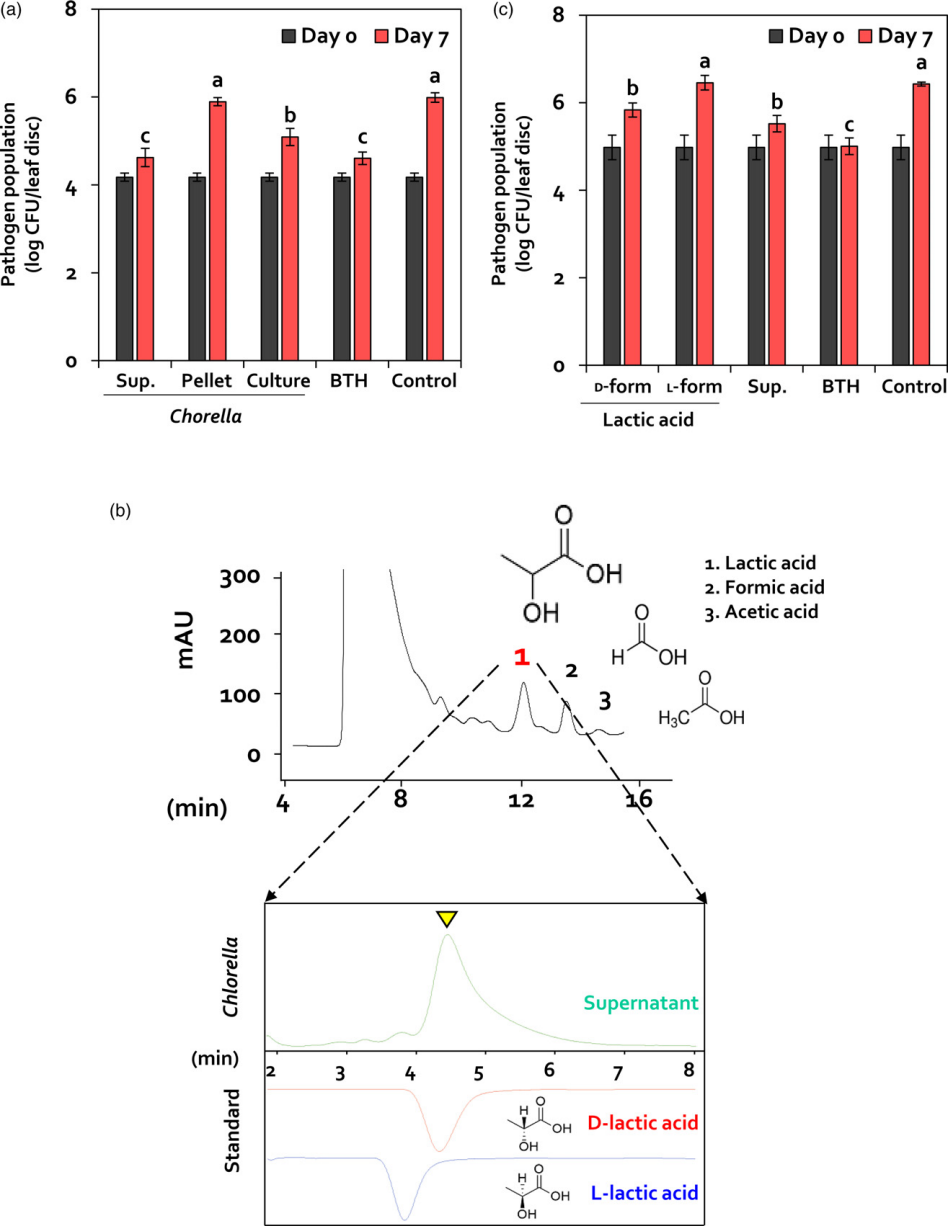
d‐Lactic acid secreted by *C. fusca* elicits induced resistance. (a) Bacterial cell count in *C. fusca* supernatant or pellet*‐*treated Arabidopsis at 0 and 7 dpi*.* Sup., filtered supernatant of mixotrophic *C. fusca*; Pellet, cell pellet of mixotrophic *C. fusca*; Culture, mixotrophic *C. fusca* culture; BTH, 0.33 mm BTH; Control, BG11 broth medium. (b) Three organic acid compounds were detected in the supernatant of *C. fusca*. (c) Bacterial cell count in d‐lactic acid‐treated or l‐lactic acid*‐*treated Arabidopsis at 0 and 7 dpi. d‐form, 10 nm d‐lactic acid; l‐form, 10 nm l‐lactic acid; Supernatant, filtered supernatant of mixotrophic *C. fusca*; BTH, 0.33 mm BTH; Control, distilled water. Data represent ± SEM (*n* = 12 replications per treatment). Different letters indicate significant differences between treatments (*P* < 0.05; LSD).

To investigate potential determinants of *C. fusca* that elicit induced resistance in Arabidopsis, the *C. fusca* culture was centrifuged and the cell pellet and supernatant were applied separately to Arabidopsis leaves (Figure 4a). At 7 dpi, the population density of *Pto* DC3000 in Arabidopsis leaves pre‐treated with *C. fusca* supernantant, cell pellet, and culture was 4.2 × 10^4^, 7.8 × 10^5^, and 1.2 × 10^5^ cfu per leaf disc, respectively, whereas pathogen population density in BG11 medium‐treated Arabidopsis leaves was 9.7 × 10^5^ cfu per leaf disc (Figure 4a). Interestingly, induced resistance against *Pto* DC3000 was also elicited in Arabidopsis leaves treated with the autoclaved supernatant of *C. fusca* culture (Figure S4). This suggests that induced resistance is elicited by a heat‐stable and secreted determinant(s) present in the *C. fusca* supernatant.

To identify *C. fusca* determinant(s) in the supernatant, fractionation of the supernatant was conducted using high‐performance liquid chromatography (HPLC) and nuclear magnetic resonance (NMR) analyses (Figure 4b). Three organic compounds, including lactic acid, formic acid, and acetic acid were identified in the hydrophilic fraction of the supernatant. Among these three compounds, lactic acid showed the highest value of milli‐absorbance units (mAU) at 12 min (Figure 4b). As lactic acid is the major compound derived from *Chlorella* species during fermentation (Syrett and Wong, 1963; Begum and Syrett, 1970), we hypothesized that lactic acid functions as the main determinant in *C. fusca*‐mediated induced resistance against *Pto* DC3000.

To assess induced resistance triggered by lactic acid, serially diluted dl‐form of lactic acid, representing the racemic mixture of d‐ and l‐lactic acid, was applied onto Arabidopsis leaves (Figure S5). Interestingly, the population density of *Pto* DC3000 in leaves pre‐treated with 10 nm dl‐lactic acid was 3.2 × 10^6^ cfu per leaf disc at 7 dpi, whereas that in the control sample was 1.3 × 10^7^ cfu per leaf disc (Figure S5). Additionally, 10 nm dl‐lactic acid did not directly inhibit the growth of *Pto* DC3000 on 1/10 King’s B agar plate (Figure S6). These results suggest that dl‐lactic acid is a putative determinant in *C. fusca* supernatant that elicits induced resistance in Arabidopsis against *Pto* DC3000.

### d‐Lactic acid secreted by *Chlorella* acts as a defence priming determinant

Previous studies have shown that *C. pyrenoidosa* and *C. vulgaris* produce d‐lactate from pyruvate by the action of d‐lactate dehydrogenase (Gruber *et al.*, 1974). Based on this report, we analyzed the stereoisomeric structure of lactic acid secreted by *C. fusca* using HPLC (Figure 4b). The *C. fusca* supernatant contained only the stereoisomeric d‐form of lactic acid (Figure 4b). Therefore, we hypothesized that d‐lactic acid specifically triggers induced resistance in Arabidopsis. To investigate this hypothesis, Arabidopsis leaves were treated separately with 10 nm each of d‐lactic acid and l‐lactic acid (Figure 4c). At 7 dpi, the population density of *Pto* DC3000 in Arabidopsis leaves pre‐treated with 10 nm d‐lactic acid, l‐lactic acid, and BG11 medium was 6.3 × 10^5^, 3.2 × 10^6^, and 2.5 × 10^6^ cfu per leaf disc, respectively (Figure 4c). These results suggest that induced resistance triggered by lactic acid is dependent on its stereoisomermic structure, and d‐lactic acid is the most likely determinant of *C. fusca*‐triggered induced resistance in Arabidopsis.

**Figure 5:**
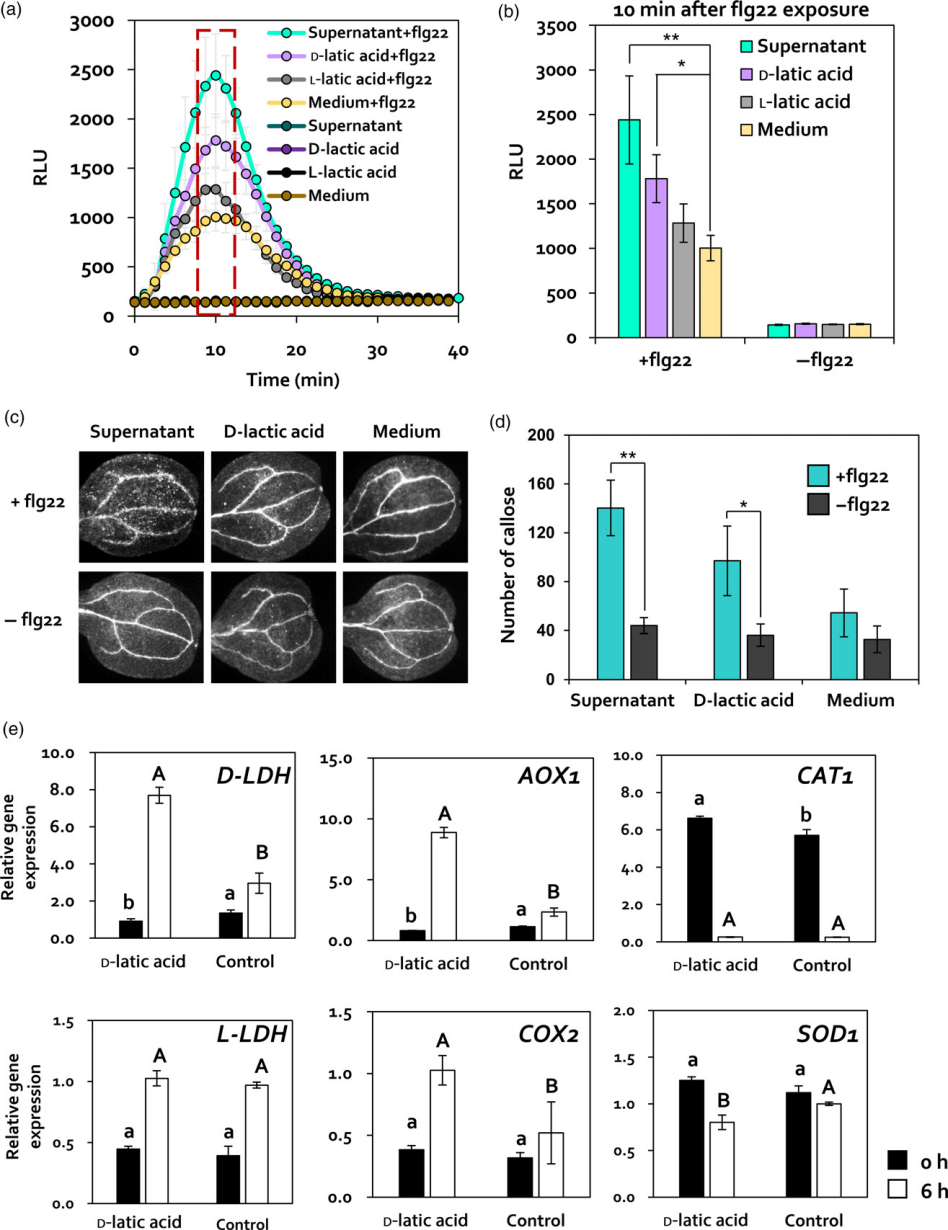
Elicitation of PTI priming by d‐lactic acid. (a, b) Quantification of ROS burst in d‐ or l‐lactic acid‐treated Arabidopsis upon flg22 (100 nm) treatment. ROS production in leaf discs of 4‐week‐old Arabidopsis plants (represented as RLU) was evaluated for 40 min. Maximum RLU signal was detected at 10 min after flg22 exposure. Data represent mean ± SE of three independent biological replicates, each consisting of six leaf discs (*n* = 18 replications per treatment). Asterisks indicate significant differences (*P < 0.05, **P < 0.001, ***P < 0.0001). Supernatant + flg22, *C. fusca* supernatant‐treated Arabidopsis with flg22; d‐lactic acid + flg22, 10 nM d‐lactic acid‐treated Arabidopsis with flg22; l‐lactic acid + flg22, 10 nm d‐lactic acid‐treated Arabidopsis with flg22; Medium + flg22, BG11 medium control with flg22; Supernatant, *C. fusca* supernatant‐treated Arabidopsis without flg22; d‐lactic acid, 10 nm d‐lactic acid‐treated Arabidopsis without flg22; l‐lactic acid, 10 nm l‐lactic acid‐treated Arabidopsis without flg22; Medium, BG11 medium control without flg22. (c, d) Callose deposition in *Chlorella* supernatant (Supernatant), 10 nm d‐lactic acid (d‐lactic acid), or BG11 medium (Medium)‐treated Arabidopsis seedlings upon 100 nm flg22 treatment. Cotyledons were collected at 1 h after flg22 exposure for aniline blue staining. Data represent the mean ± SE of three independent biological replicates (*n* = 18). Asterisks indicate significant differences (*P < 0.05, **P < 0.001, ***P < 0.0001). +flg22, 1 h after flg22 treatment; −flg22, before flg22 treatment. (e) Expression patterns of *D‐LDH*, *L‐LDH*, *AOX1*, *COX2*, *CAT1*, and *SOD1* in d‐lactic acid or sterilized distilled water (control)‐treated Arabidopsis plants at 0 and 6 h after 100 nm flg22 treatment. Different letters indicate significant differences between treatments (*P* < 0.05; LSD).

To validate whether d‐lactic acid affects PTI priming in Arabidopsis leaves, ROS burst and callose deposition were detected in Arabidopsis leaves treated with *C. fusca* superanatant, 10 nm d‐lactic acid, 10 nm l‐lactic acid, and BG11 medium using a luminol assay and aniline blue staining upon exposure to flg22 (Figure 5a–d). No RLU signal was observed in Arabidopsis leaves treated with *C. fusca* superanatant (Supernatant), 10 nm d‐lactic acid (d‐lactic acid), 10 nm l‐lactic acid (l‐lactic acid), and BG11 medium (Medium) during the detection period (Figure 5a,b). At 10 min after flg22 exposure, the RLU signal was 2.43‐ and 1.78‐fold higher in leaves treated with *C. fusca* supernatant and subsequently treated with flg22 (Supernatant + flg22) and with d‐lactic acid and subsequently treated wih flg22 (d‐lactic acid + flg22), respectively, than in treatment with BG11 medium control and subsequently treated wih flg22 (Medium + flg22); however, no difference was observed in the RLU signal between l‐lactic acid + flg22 and Medium + flg22 treatments (Figure 5b). Consistent with the results of ROS burst, callose deposition in Supernatant + flg22 and d‐lactic acid + flg22 was 2.6‐fold and 1.8‐fold higher, respectively, than in the Medium + flg 22 (Figure 5c,d). Collectively, these data indicated that foliar application of d‐lactic acid primes flg22‐triggered PTI responses in Arabidopsis.

In *Drosophila*, exogenous l‐lactic acid activates ROS production via the activation of L‐lactate oxidation by L‐lactate dehydrogenase (*L‐LDH*) in mitochondria (Iatsenko *et al.*, 2018). To investigate how exogenous d‐lactic acid affects ROS production in Arabidopsis, we evaluated the expression of mitochondrial lactate dehydrogenase genes (*D‐LDH* and *L‐LDH*) and two mitochondrial antioxidant enzyme‐encoding genes, *alternative oxidase 1* (*AOX1*) and *cytochrome C oxidase subunit 2* (*COX2*). We compared the expression level of these genes with that of cysotolic antioxidant enzyme‐encoding genes such as *catalase 1* (*CAT1*) and *superoxide dismutase 1* (*SOD1*) in leaves treated with d‐lactic acid at 0 and 12 h after flg22 treatment (Figure 5e). The expression levels of *D‐LDH*, *COX2,* and *AOX1* oxidase were significantly upregulated in d‐lactic acid‐treated Arabidopsis leaves (d‐lactic acid) by 2.66‐fold, 3.87‐fold, and 2.04‐fold, respectively, compared with BG11 medium‐treated leaves (Control) at 12 h after flg22 exposure (Figure 5e). However, no difference in *L‐LDH*, *CAT1*, and *SOD1* expression levels was detected between d‐lactic acid and BG11 medium‐treated leaves (Figure 5e). Taken together, our results suggested that exogenous d‐lactic acid activates mitochondrial *D‐LDH* expression and ROS generation. Taken together, these results suggested that d‐lactic acid secreted by *C.* *fusca* is a potential determinant of induced resistance and PTI priming in Arabidopsis.

## Discussion

*Chlorella*‐mediated biological control against plant pathogens has been demonstrated in various crop species (Bileva, 2013; Kim *et al.*, 2014; Lee *et al.*, 2016; Kim *et al.*, 2018). Nonetheless, application of *Chlorella* as a biocontrol agent in agicultural crops was not biologically relevant because *Chlorella* is primarily an aquatic microalga and, generally, does not inhabit soil. However, certain *Chlorella* species have been detected in soil and on plant surface (Treves *et al.*, 2016; Zhu *et al.*, 2018). The ubiquitous nature of *Chlorella* led us to investigate the interaction between the model plant Arabidopsis and *C. fusca*. In this study, we demonstrated the mechansims of biological control by *C. fusca* in Arabidopsis. First, foliar application of *C. fusca* protected Arabidopsis against a bacterial pathogen *Pto* DC3000 by activating plant systemic immunity, referred to as induced resistance. Second, treatment of *C. fusca* primed plant innate immunity, PTI responses in Arabidopsis. Third, d‐lactic acid in *C. fusca* supernatant was identified as a determinant of induced resistance and PTI priming.

Generally, defence priming refers to a physiological state elicited by an inducing agent that activates faster and stronger defence responses under subsequent pathogen attack (Conrath *et al.*, 2002; Conrath *et al.*, 2006). The priming agent β‐amino butyric acid (BABA) has been intensively investigated in Arabidopsis (Zimmerli *et al.*, 2000; Zimmerli *et al.*, 2001; Ton and Mauch‐Mani, 2004). Treatment of BABA primed the accumulation of *PR1* mRNA and callose deposition in Arabidopsis upon subsequent infection of bacterial pathogen *Pto* DC3000, pathogenic oomycete *Hyaloperonospora parasitica,* and pathogenic fungi including *Botrytis cinerea, Plectosphaerella cucumerina*, and *Alternaria brassicicola* (Zimmerli *et al.*, 2000; Zimmerli *et al.*, 2001; Ton and Mauch‐Mani, 2004). Additionally, BABA‐treated Arabidopsis plants also showed enhanced expression of a PTI marker gene, *FRK*, and greater callose deposition after exposure to the bacterial PAMP flg22 (Singh *et al.*, 2012). The enhanced PTI response in BABA‐treated plants was reported as ‘PTI priming’ (Singh *et al.*, 2012). In Arabidopsis, PTI priming is associated with receptor kinases such as *LecRK‐VI.2* and *CRK* (Singh *et al.*, 2012; Huang *et al.*, 2014; Yeh *et al.*, 2015; Lee *et al.*, 2017). *CRK4*, *CRK6*, and *CRK36* funtion as positive regulators of PTI priming (Yeh *et al.*, 2015; Lee *et al.*, 2017). Because these three genes positively affected *C. fusca*‐triggered induced resistance against *Pto* DC3000 in Arabidopsis (Figure 2c,d), we hypothesized that *C. fusca* treatment induces PTI priming (Figure 3). Unlike PTI priming by BABA treatment (Singh *et al.*, 2012), *C. fusca* and its determinant d‐lactic acid primed not only callose depostion but also ROS burst (Figure 3). Apoplastic ROS play important roles in callose deposition and cell wall cross‐linking to impede the penetration of pathogens (Daudi *et al.*, 2012; O’Brien *et al.*, 2012; Qi *et al.*, 2017). The lack of callose deposition in peroxidase knockdown mutants *fbp1.1* and *prx34* was rescued by exogenous supply of hydrogen peroxide (Daudi *et al.*, 2012). In addition, hydrogen peroxide activated the expression of *MYB51*, *CYP79B2*, and *CYP81F2* genes, which are required for callose deposition (Daudi *et al.*, 2012). Therefore, our results indicated that *C. fusca* and d‐lactic acid sequentially primed ROS burst and callose deposition.

Previously, ROS production by l‐lactic acid has been reported in human leukocytes and *Drosophila* intestinal cells (Echigoya *et al.*, 2012; Iatsenko *et al.*, 2018). l‐Lactic acid triggered NADPH oxidase (Nox)‐mediated ROS production through L‐lactate oxidation by the mitochondrial L‐LDH in *Drosophila* intestinal cell (Iatsenko *et al.*, 2018). In plants, the role of mitochondrial LDH on plant defence and ROS production is largely unexplored. Diverse plant species such as Arabidopsis, rice, and *Jerusalem artichoke* possess not only L‐LDH, but also D‐LDH, which oxidizes d‐lactate (Atlante *et al.*, 2005; Welchen *et al.*, 2016; An *et al.*, 2017). Based on previous reports on ROS production by l‐lactic acid in animal cells, we hypothesized that exogenous d‐lactic acid induces ROS production in Arabidopsis via the oxidation of d‐lactate by d‐LDH. Indeed, exogenous d‐lactic acid activated the expression of *D‐LDH* gene in Arabidopsis after flg22 treatment (Figure 5e). *In planta*, d‐lactate is oxidized by d‐LDH in mitochondrial intermembrane space, and electrons‐derived d‐lactate oxidation are delivered to the respiratory chain through cytochorome *c* (Atlante *et al.*, 2005; Welchen *et al.*, 2016). The expression of *COX2* and *AOX1*, which encode antioxidants located in the mitochondrial membrane (Rhoads *et al.*, 2006), was activated in Arabidopsis by d‐lactic acid after flg22 treatment (Figure 5e). However, the expression of genes encoding CAT1 and SOD1 enzymes, which are largely located in peroxisomes and cytosol, respectively (Weydert and Cullen, 2010), showed no differences between d‐lactic acid‐treated and untreated Arabidopsis plants (Figure 5e). The mitochondrial antioxidant enzymes might be activated to catalyze mitochondrial ROS production in d‐lactic acid pre‐treated Arabidopsis by subsequent treatment of flg22. Therefore, our results suggest that exogenous d‐lactic acid induces the generation of mitochondrial ROS through plant‐specific d‐lactate oxidation in response to PAMP molecules in Arabidopsis. Taken together, our results suggest that d‐lactic acid in *C. fusca* functions as a PTI priming agent to enhance ROS production in Arabidopsis via the activation of d‐lactate oxidation in mitochondria.

The supernatant of xenic *Chlorella* cultures contained three organic compounds: lactic acid, formic acid, and acetic acid (Figure 4b). Previous studies identified these organic acids as major products of *Chlorella* species in the presence of glucose (Syrett and Wong, 1963; Begum and Syrett, 1970). Because these organic acid compounds are also produced by bacteria in xenic *Chlorella* cultures, it is difficult to identify *Chlorella*‐derived organic acids. However, among these three organic acids, only lactic acid has been identified as a *Chlorella*‐specific compound, based on stereoismeric structure analysis (Gruber *et al.*, 1974). In nature, lactic acid exists in three forms: dl, l, and d (Maurino and Engqvist, 2015). Animals, human, and bacteria mainly produce the l‐ or dl‐form (Maurino and Engqvist, 2015). The d‐lactic acid biosynthesis pathway is largely unknown, except in *Leuconostoc* spp., *Lactobacillus* spp., and *Pediococcus* spp. (Carr *et al.*, 2002). However, *Chlorella* spp. can produce only d‐lactic acid from glucose by the action of d‐LDH (Gruber *et al.*, 1974). Because we did not identify *Leuconostoc* spp., *Lactobacillus* spp., and *Pediococcus* spp. among *C. fusca*‐associated bacteria (Figure 1d), d‐lactic acid in the supernatant of *C. fusca* xenic culture was most likely to have been produced by *C. fusca* (Gruber *et al.*, 1974). Additionally, previous reports of reduction in LDH activity in the dark (Perez‐Garcia and Bashan, 2015) support our conclusion that d‐lactic acid in mixotrophic *Chlorella* culture is a determinant of induced resistance in Arabidopsis (Figure 1b). Interestingly, induced resistance and ROS production were activated by lactic acid in a stereoisomer‐dependent manner; Arabidopsis leaves did not respond to l‐lactic acid but responded to d‐lactic acid (Figure 4c). Conversely, ROS production was activated only by l‐lactic acid in animal cells (Iatsenko *et al.*, 2018). This difference between plant and animal systems may be due to the presence and expression of *D‐LDH* genes in plants, unlike animals (Iatsenko *et al.*, 2018; Monroe *et al.*, 2019). The *D‐LDH* genes are conserved in higher plants and green algae, including *Chlorella* spp. (Gruber *et al.*, 1974; Atlante *et al.*, 2005; Welchen *et al.*, 2016; An *et al.*, 2017), but are absent in animals, including human and *Drosophila* (Iatsenko *et al.*, 2018; Monroe *et al.*, 2019). Therefore, our results suggested that d‐lactic acid might be a conserved signalling molecule in photosynthetic organisms including *Chlorella* spp. and land plants including angiosperms.

To investigate the molecular basis of defence priming by *Chlorella*‐mediated induced resistance, we focused on DEGs upregulated in *C. fusca*‐treated Arabidopsis leaves at 12 hpi (Figure 2), based on previous reports (Verhagen *et al.*, 2004; Stringlis *et al.*, 2018). The most striking feature of our transcriptome data was the identification of the expression of the *CRK* family genes, which were highly upregulated in *C. fusca*‐treated Arabidopsis leaves (Figure 2b,c). The *CRK* genes represent a large subgroup of receptor‐like kinases (RLKs) and harbour a C‐X8‐C‐X2‐C (DUF26) motif with unknown function in the extracellular domain (Chen, 2001). Additionally, *CRK* genes play critical roles in biotic and abiotic stress resistance in plants (Chen, 2001; Yeh *et al.*, 2015; Lee *et al.*, 2017). The *CRK4, CRK6*, and *CRK36* genes positively regulate PTI responses and enhance resistance against *Pto* DC3000 in Arabidopsis (Yeh *et al.*, 2015; Lee *et al.*, 2017). *C. fusca* treatment primed the expression of *CRK4, CRK6,* and *CRK36* in Arabidopsis after infection with *Pto* DC3000 (Figure 2b,c). Thus, our results suggest a correlation between PTI priming and *CRK* activation in *C. fusca*‐treated Arabidopsis. *CRK* genes, including *CRK2* and *CRK3,* activate many transcription factors such as WRKY DNA‐binding proteins (Nemoto *et al.*, 2015). In our study, genes encoding WRKY group III proteins with an altered C2‐HC zinc finger motif (C‐X7‐C‐X23‐HX‐C) (Kalde *et al.*, 2003), such as *WRKY53, WRKY54,* and *WRKY70,* were upregulated in *C. fusca*‐treated Arabidopsis leaves (Figure 2c). WRKY group III proteins act as key components of biotic stress responses and SA and JA signalling (Li *et al.*, 2004; Wang *et al.*, 2006). This situation is consistent with our results; we showed that *C. fusca*‐elicited induced resistance in Arabidopsis was simutaneously regulated by SA and JA signalling at 12 hpi (Figure 2c,e). The synergistic effect of SA and JA signalling on plant immunity has been previously reported in Arabidopsis, rice, potato, and tobacco (van Wees *et al.*, 2000; Halim *et al.*, 2009; Tamaoki *et al.*, 2013; Zhu *et al.*, 2014). Taken together, the transcriptome data suggested that *C. fusca* treatment primes the expression of defence signalling genes, including *CRK* and *WRKY*, and hormone signalling genes in *Arabidospsis*.

**Figure 6:**
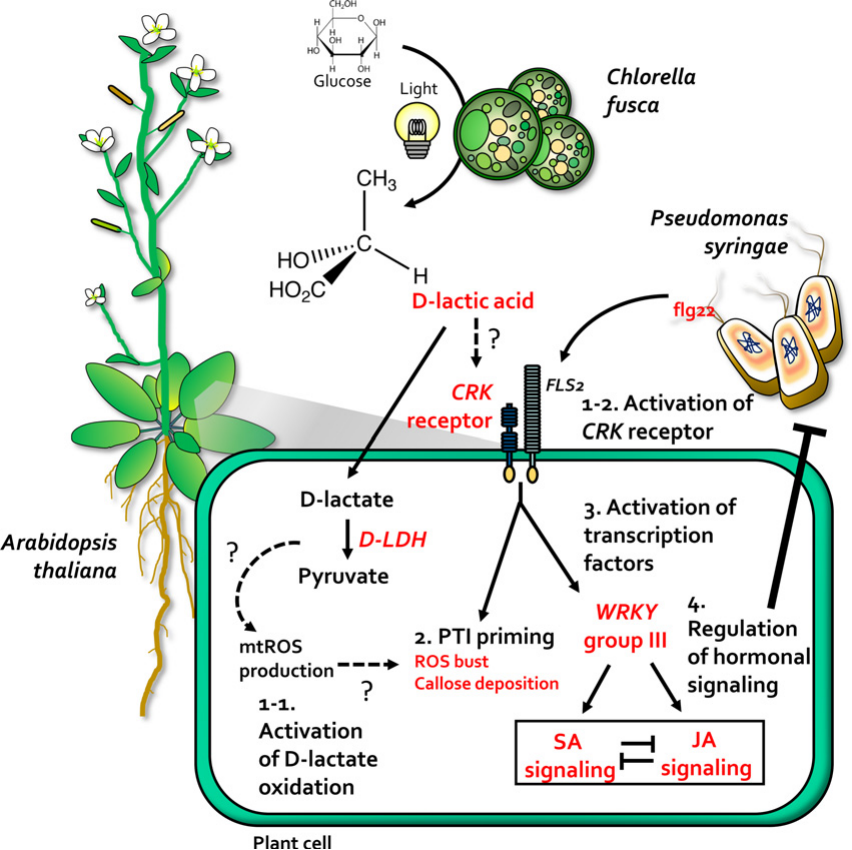
A model depicting the mechanism of *C. fusca* eliciting plant immunity against *Pto* DC3000. *Chlorella fusca* secretes d‐lactic acid in the presence of glucose and light. (1‐1) Application of *C. fusca* primes the expression of *CRK* receptor gene, which positively regulates PAMP‐triggered immunity (PTI) response upon flg22 exposure or *Pto* DC3000 infection. (1‐2) *C. fusca*‐derived d‐lactic acid primes *D‐LDH* expression, leading to the oxidation of d‐lactate, which in turn induces mitochondrial ROS (mtROS) production upon flg22 exposure or *Pto* DC3000 infection. (2) PTI priming might be induced by the activation of d‐lactate oxidation and *CRK* expression by *C. fusca* treatment. (3) Additionally, the CRK receptor can activate transcription factors, especially WRKY group III. (4) Activation of *WRKY* group III genes regulates salicylic acid and jasmonic acid signalling. Therefore, *C. fusca* elicits plant immunity against *Pto* DC3000 by orchestrating a series of defence mechanisms.

Overall, we demonstrated the mechanism of biological control employed by the d‐lactic acid‐secreting *C. fusca* to trigger induced resistance and PTI primng in Arabidopsis (Figure 6). Like BABA, d‐lactic acid was identified as a PTI priming agent in *C. fusca*. Because *C. fusca* treatment enhanced PTI responses, the application of *Chlorella* species can activate broad‐spectrum resistance in diverse crop species (Huang and Zimmerli, 2014). Commercial uses of *Chlorella* species as biofuels and health supplements are mostly based on cells, which lead to economical and environmental problems because of the filtration and disposal processes involved in obtaining large amounts of supernatant after harvesting the cells. For example, to obtain 10 000 tons of biodiesel per year, *Chlorella* supernatant produced from a 150 ton bioreactor is disposed of (Tabernero *et al.*, 2012). Therefore, recycling the *Chlorella* supernatant as a biological control agent in the agricultural sector can be very economical and sustainable. However, to commercialize *Chlorella* supernatant as a biological control agent, it is necessary to concentrate the supernatant from several hundered tons of culture. In the industrial sector, d‐lactic acid can be used as a quality control marker for large‐scale field applications of the supernatant. This study is the first demonstration of the molecular mechanism underlying the *Chlorella*–higher plant interaction. Our results will help to broaden the agricultural applications of *Chlorella* by supernatant recycling.

## Experimental Procedures

### Plant materials

*Arabidopsis thaliana* (L.) Heynh. ecotype Columbia (Col‐0; wild‐type), *crk 4*, *crk 6*, *npr1*, and *jar1* mutants, and the *NahG* overexpression line were used in this study (Zipfel *et al.*, 2004). The *crk4* and *crk6* mutants were gifted by Michael Wrzaczek at the University of Helsinki, Helsinki, Finland. Seeds were surface sterilized with 6% sodium hypochlorite for 10 min, washed five times with sterile distilled water, and then placed on half‐strength Murashige and Skoog medium supplemented with 0.6% (w/v) agar and 1.5% (w/v) sucrose. The plates were incubated at 21°C under a 16 h light/8 h dark cycle for 7 days. Seedlings were transplanted in soil‐less potting medium (Punon Horticulture Nursery Medium Low; Punong Co., Ltd., Gyeongju, Korea) and grown in an environmentally controlled growth room at 25°C under fluorescent lights (*c.* 7000 lux light intensity) and a 12 h light/12 h dark cycle.

### Cultivation and application of *C. fusca*

*C. fusca* strain CHK0059 was obtained from Chang‐Ki Shim at the Rural Development Administration, Wanju, South Korea, and grown under three different conditions (mixotrophic, heterotrophic, or autotrophic) in BG11 broth (Rippka *et al.*, 1979). Under mixotrophic conditions, 1 g glucose was added to 1 L BG11 medium. Heterotrophic cultivation of *C. fusca* was carried out in the dark. Under all three growth conditions, *C. fusca* was grown at 25°C and under continuous illumination (100 µmol m^−2^ sec^−1^) in a 1 L photobioreactor containing 800 ml of autoclaved BG11 broth. To calculate the concentration of *C. fusca*, cell numbers were counted using a haemocytometer (INCYTO, Cheonan, Korea). When the concentration reached 10^7^ cells ml^−1^ (exponential phase), cells were harvested and centrifuged at 4000 ***g*** for 10 min to separate the supernatant from the pellet. To obtain the axenic *C. fusca* culture, colonies were picked on R2A agar plates containing 50 μg ml^−1^ kanamycin, 1 μg ml^−1^ polymyxin B, and 5 μg ml^−1^ vancomycin, until the colonies were bacteria‐free.

To examine plant immunity induced by *C. fusca*, Arabidopsis leaves were sprayed with 20 ml of 10^7^, 10^5^, or 10^3^ cells ml^−1^ *C. fusca* culture, 0.33 mm BTH, or BG11 broth at 1 and 2 weeks after transplantation of Arabidopsis seedlings. To identify the determinant of induced resistance in *C. fusca*, the cell pellet and supernatant were separated from the mixotrophic *C. fusca* culture (10^7^ cells ml^−1^) and applied to Arabidopsis leaves separately. The supernatant from *C. fusca* was filtered using a 0.45‐µm syringe filter.

### Inoculation of pathogen and quantification of pathogen population

*P. syringae* pv. *tomato* DC3000 (spontaneous rifampicin‐resistant strain) was grown on King’s B agar plates containing 100 µg ml^−1^ rifampicin at 30°C for 2 days. At 7 days after application of *C. fusca*, *P. syringae* pv. *tomato* DC3000 was suspended in distilled water. The optical density of the suspension was measured at an absorbance of 600 nm (OD_600_) using a spectrophotometer (Biochrom US, MA, USA). A pathogen suspension (OD_600_ = 1.0) was sprayed on Arabidopsis leaves. To determine pathogen population, *C. fusca*‐pre‐treated leaf discs (diameter = 0.8 cm) were harvested at the designated time points at 0 and 7 dpi and ground immediately in 10 mm MgCl_2_. The pathogen was plated on King’s B agar plates containing 100 µg ml^−1^ rifampicin and incubated at 30°C. The pathogen population was determined after 3 days.

### Scanning electron microscopy

To investigate symbiotic bacteria on the surface of *C. fusca*, samples were fixed in 2.5% glutaraldehyde in 1× phosphate‐buffered saline (PBS) for 10 min and post‐fixed in 1% osmium tetroxide in the same buffer for 1 h. Subsequently, samples were dehydrated using a graded ethanol series and substituted by hexamethyldisilazane. Samples were then dried and observed under a scanning electron microscope (HITACHI S4800; Hitachi, Tokyo, Japan).

### RNA extraction and RNA‐seq experiments

Two leaves per plant were collected from 12 Arabidopsis plants at 0 and 12 hpi. To minimize stress‐induced gene expression, intact leaves were selected and immediately frozen in liquid nitrogen. Total RNA was isolated from leaves using TRIzol Reagent (Molecular Research Inc., OH, USA), according to the manufacturer's instructions as described previously (Choi *et al.*, 2014). The quality and integrity of total RNA were confirmed by agarose gel electrophoresis and NanoDrop spectrophotometry (Thermo Fisher Scientific Inc., DE, USA). To conduct RNA‐seq, a sequencing library was prepared using the TruSeq RNA Sample Preparation Kit v2 (Illumina, CA, USA), according to the manufacturer’s instructions. Briefly, mRNA was purified from total RNA using poly‐T oligo‐attached magnetic beads. The purified mRNA was fragmented and converted to cDNA. Adapters were then ligated to the cDNAs and the resulting fragments were amplified by PCR. RNA‐seq was performed using a HiSeq‐2000 instrument (Illumina) to generate paired‐end reads (2 × 100 bp).

### RNA‐seq data processing and *in silico* analysis

Arabidopsis* thaliana* reference genome sequence (v.10) was obtained from The Arabidopsis Information Resource (TAIR; assembly ID: TAIR10). Reference genome index construction and read mapping were performed using STAR (v.2.5.1b). The RNA‐seq data were deposited in the NCBI Gene Expression Omnibus public database under accession number GSE89894. To determine gene expression, counts per million (CPM) mapped reads of each sample were calculated. The CPM data were normalized using quantile normalization in the R language environment (v.3.2.5). The measured gene expression values were log_2_‐transformed and median‐centred across genes and samples. To assess differences in gene expression among samples, scatterplot matrices were plotted using the Pearson correlation test method. Gene set enrichment analysis was carried out to identify the most significant gene sets associated with the GO biological process or Kyoto Encyclopedia of Genes and Genomes (KEGG) pathways, and the significance of over‐represented gene sets was estimated using Fisher’s exact test. Gene set enrichment analysis was performed using DAVID Bioinformatics Resources (v.6.8).

### Validation of RNA‐seq data using qRT‐PCR

To validate RNA‐seq data, expression of selected DEGs (including defence marker genes *CRK*, *WRKY*, and *GST*) was analyzed by qRT‐PCR. To perform qRT‐PCR, first‐strand cDNA was synthesized from 2 mg of DNase‐treated total RNA using oligo‐dT primers and Moloney murine leukaemia virus reverse transcriptase (MMLV‐RT, Enzynomics, Daejeon, South Korea). PCR reactions were performed according to the manufacturer’s instructions. Expression of DEGs was analyzed using the primers listed in Table S1. A Chromo4 Real‐Time PCR system (Bio‐Rad, CA, USA) was used for qRT‐PCR. Reaction mixtures contained cDNA template, iQTM SYBR^®^ Green Supermix (Bio‐Rad, CA, USA) and 10 pM of each primer. Thermocycler parameters used for qRT‐PCR were as follows: initial polymerase activation at 95°C for 10 min, followed by 40 cycles of denaturation at 95°C for 30 sec, annealing at 55°C for 60 sec, and extension at 72°C for 30 sec. Conditions were determined by comparing threshold values in a series of dilutions of the reverse‐transcribed product, a non‐reverse‐transcribed template control, and a non‐template control for each primer pair. Relative RNA levels were calibrated and normalized relative to the level of *AtActin2* mRNA.

### Detection of ROS

Arabidopsis leaf discs (4 mm diameter) were transferred to water in a 96‐well black plate (SPL Life Sciences Co. Pocheon‐si, South Korea) and incubated at room temperature overnight. Before the induction of ROS, the normalized leaf discs were floated on *C. fusca* culture, *C. fusca* supernatant, 10 nm lactic acid, and BG11 broth in a 96‐well plate for 1 h and then washed twice with sterile distilled water. To trigger ROS production, leaf discs were placed in 100 μl of assay solution containing 10 ng ml^−1^ peroxidase, 20 μm luminol, and 100 nm flg22 peptide. Light emission was measured as RLU using a Mithras Tristar2 LB 940 (Berthold Technologies GmbH & Co. KG, Bad Wildbad, Germany), which is a 96‐well luminometer, for 1 h.

### Callose deposition assay

Arabidopsis seedlings were grown on half‐strength Murashige and Skoog medium supplemented with 0.6% (w/v) agar and 1.5% (w/v) sucrose at 21°C and a 16 h light/8 h dark cycle for 2 weeks. Arabidopsis seedlings were incubated in *C. fusca* culture, *C. fusca* supernatant, 10 nm lactic acid, and BG11 broth in a 50 ml tube at room temperature overnight. Subsequently, Arabidopsis seedlings were washed twice with sterile distilled water. To trigger callose deposition, Arabidopsis seedlings were incubated for 1 h in 100 nm flg22. To trigger callose deposition by live *P. syringae*, Arabidopsis seedlings were incubated in the cell suspension from the wild‐type or *hrcC* ^−^ mutant of *Pto* DC3000 (OD_600_ = 1). After exposure to flg22 or *P. syringae*, Arabidopsis seedlings in 50 ml Falcon tubes were de‐stained with 95% ethanol overnight. After de‐staining, the cotyledons were detached and incubated in 150 mm K_2_HPO_4_ for 30 min. Then, the cotyledons were placed in 50 ml Falcon tubes containing 150 mm K_2_HPO_4_/0.01% aniline blue, wrapped in aluminium foil to avoid light, and stained for 2 h. Callose in the stained cotyledons was visualized under an epifluorescence microscope (Olympus BX51; Tokyo, Japan). Callose deposition was quantified using a fluorescence microscope fitted with a 4′,6‐diamidino‐2‐phenylindole (DAPI) filter (excitation wavelength, 370 nm; emission wavelength,  509 nm). The number of stained callose depositions was counted using the ‘analyze particles’ function in ImageJ software (http://rsb.info.nih.gov/ij/).

### HPLC and NMR analysis

HPLC analysis was carried out using the L‐2455 system (Hitachi, Japan) equipped with a photodiode array detector. First, a preparative HPLC column (Cosmosil 5C18‐MS II, 10 mm × 150 mm; Nacalai Tesque, Japan) was used with a mobile phase comprising 0.04% trifluoroacetic acid in water, a flow rate of 3 ml min^−1^, and a detection wavelength of 222 nm. Then, an analysis column (TSK‐GEL ODS‐100V, 4.6 mm × 150 mm; TOSHO, Japan) was used with a mobile phase comprising 0.04% trifluoroacetic acid in water (A) and methanol (B) under the following gradient conditions: 100% A for 14 min and 100% B for 20 min. A flow rate of 1 ml min^−1^ and a detection wavelength of 224 nm were used with the analysis column. ^1^H (600 MHz) and ^13^C (150 MHz) NMR spectra were obtained using a JEOL JNM‐ECA600 600MHz FT‐NMR spectrometer, with deuterium oxide as a solvent. Qualitative analyses of *C. fusca* supernatant were conducted by HPLC. HPLC was performed on an Aminex HPX‐87 column (300 mm × 4.6 mm; particle size = 5 μm; USA) at 25°C. The flow rate was 0.6 ml min^−1^. The mobile phase was 4 mM sulfuric acid in water for a total running time of 25 min. The sample injection volume was 10 μl. The detection wavelength was 215 nm.

### Statistical analysis

Data were analyzed by analysis of variance using JMP 4.0 software (SAS Institute Inc., Cary, NC, USA). Significant treatment effects were determined on the basis of the magnitude of the F‐value (*P* < 0.05). When a significant F‐value was obtained, the separation of means was analyzed by determination of Fisher’s protected least significant difference at *P* < 0.05.

## Author Contributions

S‐ML and C‐MR designed experiments and wrote the paper. S‐ML performed the assessment of induced resistance, antagonism test, Arabidopsis mutant analysis, qRT‐PCR, determinant screening, and PTI priming test. S‐KK analyzed RNA‐seq data. N‐KL and C‐YA performed cultivation of *C. fusca* and scanning electron microscopy. All authors discussed the results.

## Conflict of Interest

The authors have no conflict of interest to declare.

## Supporting information


**Table S1.** List of primers used for qRT‐PCR analysis.

**Figure S1.** Optimization of cell density and growth conditions of *Chlorella fusca* for biological control against *Pseudomonas syringae* pv. tomato DC3000 (*Pto* DC3000) in Arabidopsis.

**Figure S2.** Induced resistance‐associated differentially expressed genes (DEGs) in *C. fusca*‐treated Arabidopsis leaves at 0 and 12 hpi.

**Figure S3.** Gene ontology (GO) enrichment analysis of DEGs identified in *C. fusca*‐treated Arabidopsis leaves at 12 hpi.

**Figure S4.** Activation of *C. fusca* supernatant‐triggered induced resistance in Arabidopsis against *Pto* DC3000 with or without heat treatment.

**Figure S5.** Optimization of dl‐lactic acid concentration for eliciting induced resistance against *Pto* DC3000 in Arabidopsis.

**Figure S6.** Analysis of antagonism between filtered *C. fusca* supernatant or dl‐lactic acid and *Pto* DC3000.

## Data Availability

RNA‐seq data used in this study are freely available at the NCBI Gene Expression Omnibus public database under accession number GSE89894 (https://www.ncbi.nlm.nih.gov/geo/query/acc.cgi?acc=GSE89894).
