# Supplementary material for: d‐Lactic acid secreted by Chlorella fusca primes pattern‐triggered immunity against Pseudomonas syringae in Arabidopsis
Source: Plant J. 2020 Jan 27;102(4):761–78. doi: 10.1111/tpj.14661 (PMC7318130; doi:10.1111/tpj.14661)
Supplement: Supplementary file 1 — Table S1. List of primers used for qRT‐PCR analysis. [file TPJ-102-761-s001.docx]

**Supplementary Table 1. List of primers used for qRT-PCR analysis.**

| **RT-PCR primers** | **Primer Sequence (5'-3')** |
| --- | --- |
| *AOX1*-F | GGAGGCTTCCTGCTGATGCGACA |
| *AOX1*-R | AGCTGGAGCTTCCTTTAGTTCACGACC |
| *CAT2*-F | CCTCGTGGTTTTGCAGTCAA |
| *CAT2*-R | TCAAACTTTCAGGGTGGTGG |
| *CHIB*-F | GCTTCAGACTACTGTGAACC |
| *CHIB*-R | TCCACCGTTAATGATGTTCG |
| *COX2*-F | TGATGCTGTACCTGGTCGTT |
| *COX2*-R | TGATTGGATACCCGAGAACC |
| *CRK4*-F | GAAAAGGCCGGAACTCCTCT |
| *CRK4*-R | AGTGAGCCCGCAGTGGTAAT |
| *CRK6*-F | GATGATATGGCAACCGCAGA |
| *CRK6*-R | CCAAAACCACCTCGACCAAT |
| *CRK36*-F | AGGAACAGTTGCCCGTCCTA |
| *CRK36*-R | CAACAACCACAACGCTCCAT |
| *D-LDH*-F | CCTTGCAGAACTCATATCAAGATC |
| *D-LDH-*R | CATGAGGAGGAATTAACTTTCC |
| GSTF6-F | CTTCGCAACCCCTTTGGTAA |
| GSTF6-R | TATGATCGCCATGTCCTTGC |
| *GSTF7*-F | ACACAGGCTTGGTGAGTCCA |
| *GSTF7*-R | ACCCAAGCACTGACATGTGG |
| *GSTU4*-F | TTGCAATCAAGGAGGCTCAA |
| *GSTU4*-R | GCGACCAAGTCCAAAAATCC |
| *L-LDH*-F | CGCAGATCATCTCGACGTTA |
| *L-LDH*-R | CATAGGCGCTACCAACAACA |
| *PR1*-F | TTCACAACCAGGCACGAGGAG |
| *PR1*-R | CCAGACAAGTCACCGCTACCC |
| *PDF1.2*-F | CACCCTTATCTTCGCTGCTC |
| *PDF1.2*-R | GTTGCATGATCCATGTTTGG |
| *SOD1*-F | CCTGAGGATGCTAATCGACA |
| *SOD1*-R | TAGCCCTGGAGACCAATGAT |
| *WRKY53*-F | CATCCTCACCGAGCGTACAA |
| *WRKY53*-R | CCTCCATCGGCAAACTCTTC |
| *WRKY54*-F | GTGGTCCAGATCCCGTTGAT |
| *WRKY54*-R | ATCCCCACATGAAGCATTCC |
| *WRKY70*-F | ACGCAGAAACTCCCAAGAGC |
| *WRKY70*-R | AAACCATTTCTGGCCACACC |
| *AtActin2*-F | GAAGAACTATGAATTACCCGATGG |
| *AtActin2*-R | TACAGATCCTTCCTGATATCCACA |
