# Supplementary figures and images for: d‐Lactic acid secreted by Chlorella fusca primes pattern‐triggered immunity against Pseudomonas syringae in Arabidopsis
Source: Plant J. 2020 Jan 27;102(4):761–78. doi: 10.1111/tpj.14661 (PMC7318130; doi:10.1111/tpj.14661)

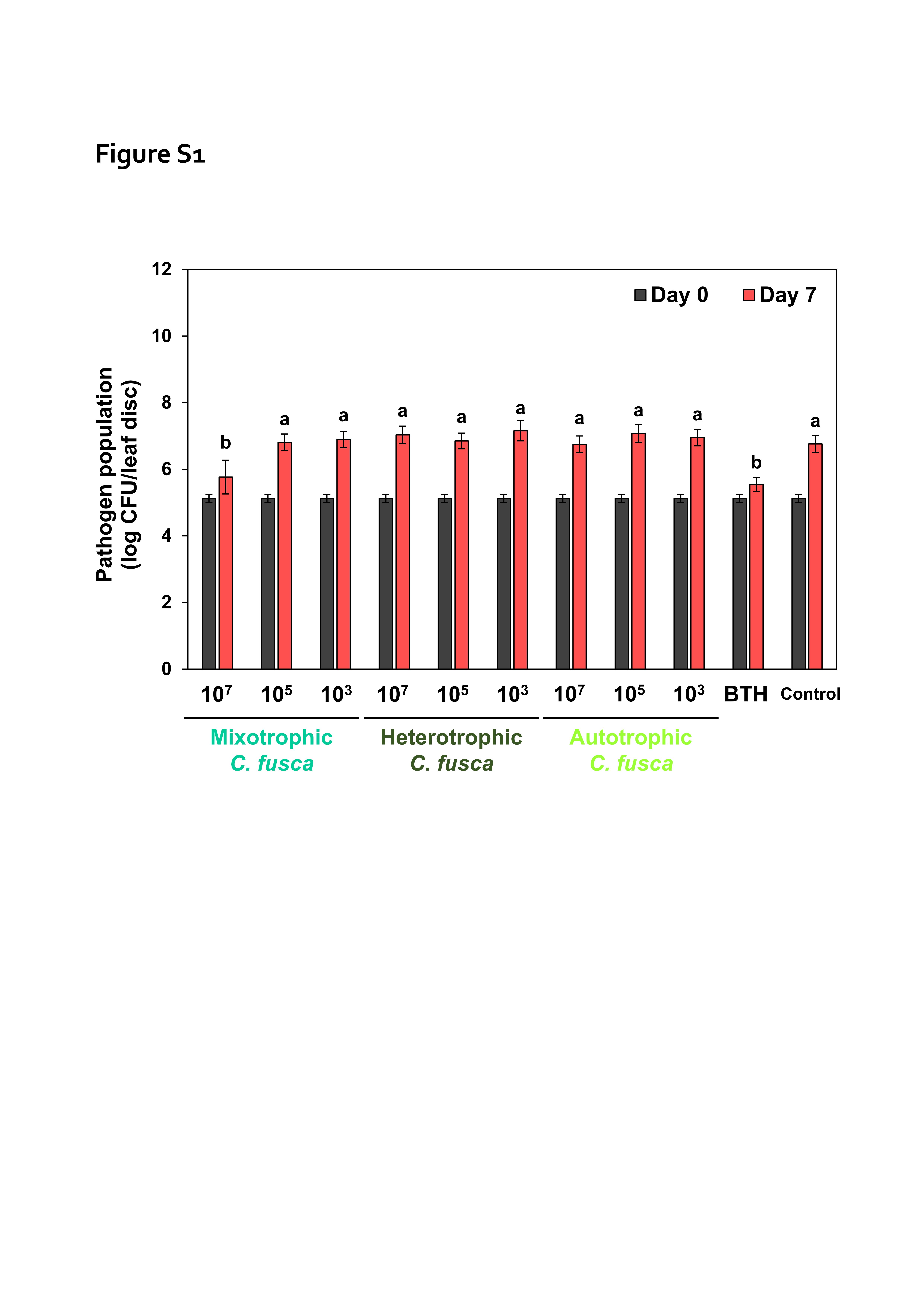

Supplement: Supplementary file 2 — Figure S1. Optimization of cell density and growth conditions of Chlorella fusca for biological control against Pseudomonas syringae pv. tomato DC3000 (Pto DC3000) in Arabidopsis. [file TPJ-102-761-s002.png]

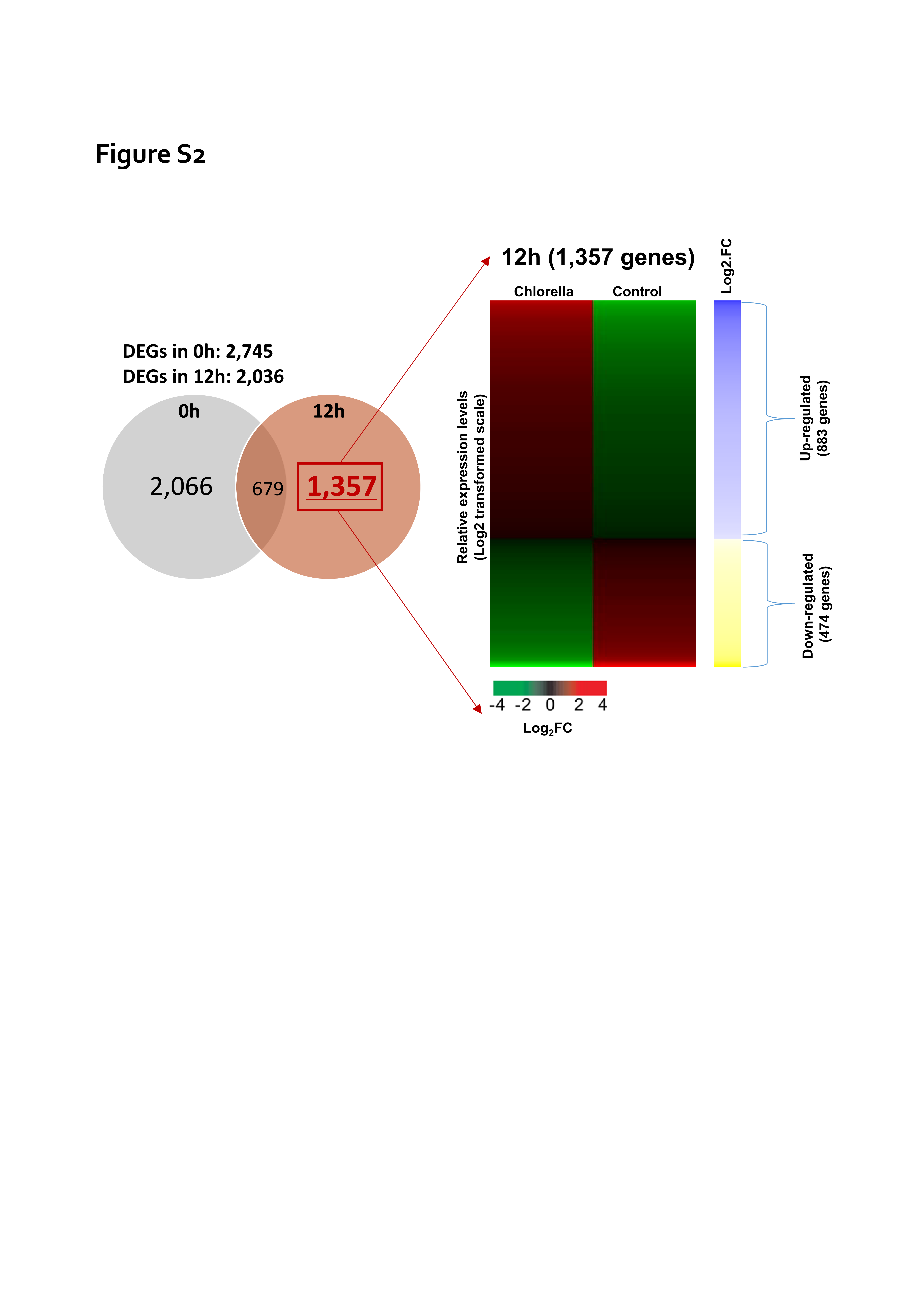

Supplement: Supplementary file 3 — Figure S2. Induced resistance‐associated differentially expressed genes (DEGs) in C. fusca‐treated Arabidopsis leaves at 0 and 12 hpi. [file TPJ-102-761-s003.png]

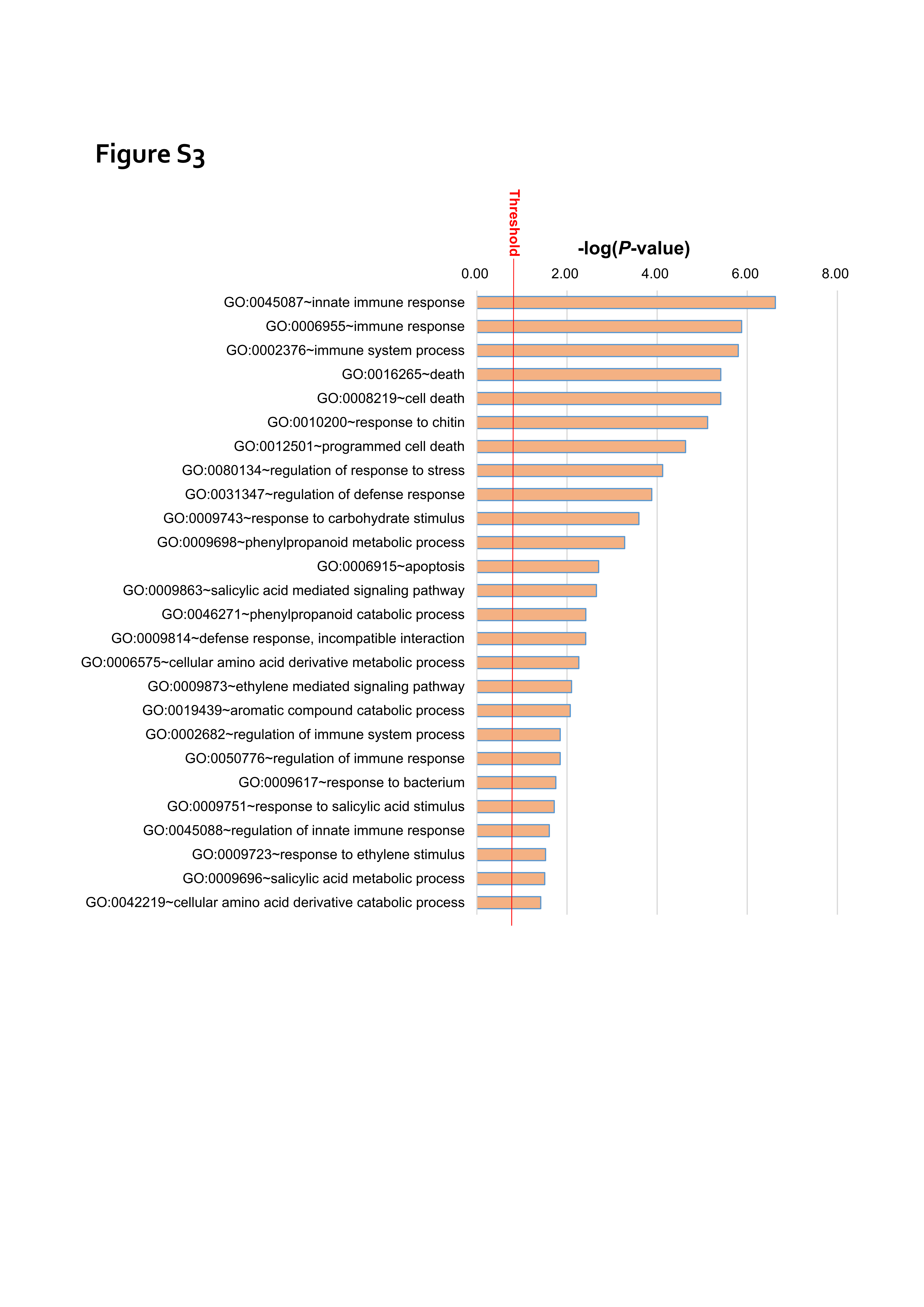

Supplement: Supplementary file 4 — Figure S3. Gene ontology (GO) enrichment analysis of DEGs identified in C. fusca‐treated Arabidopsis leaves at 12 hpi. [file TPJ-102-761-s004.png]

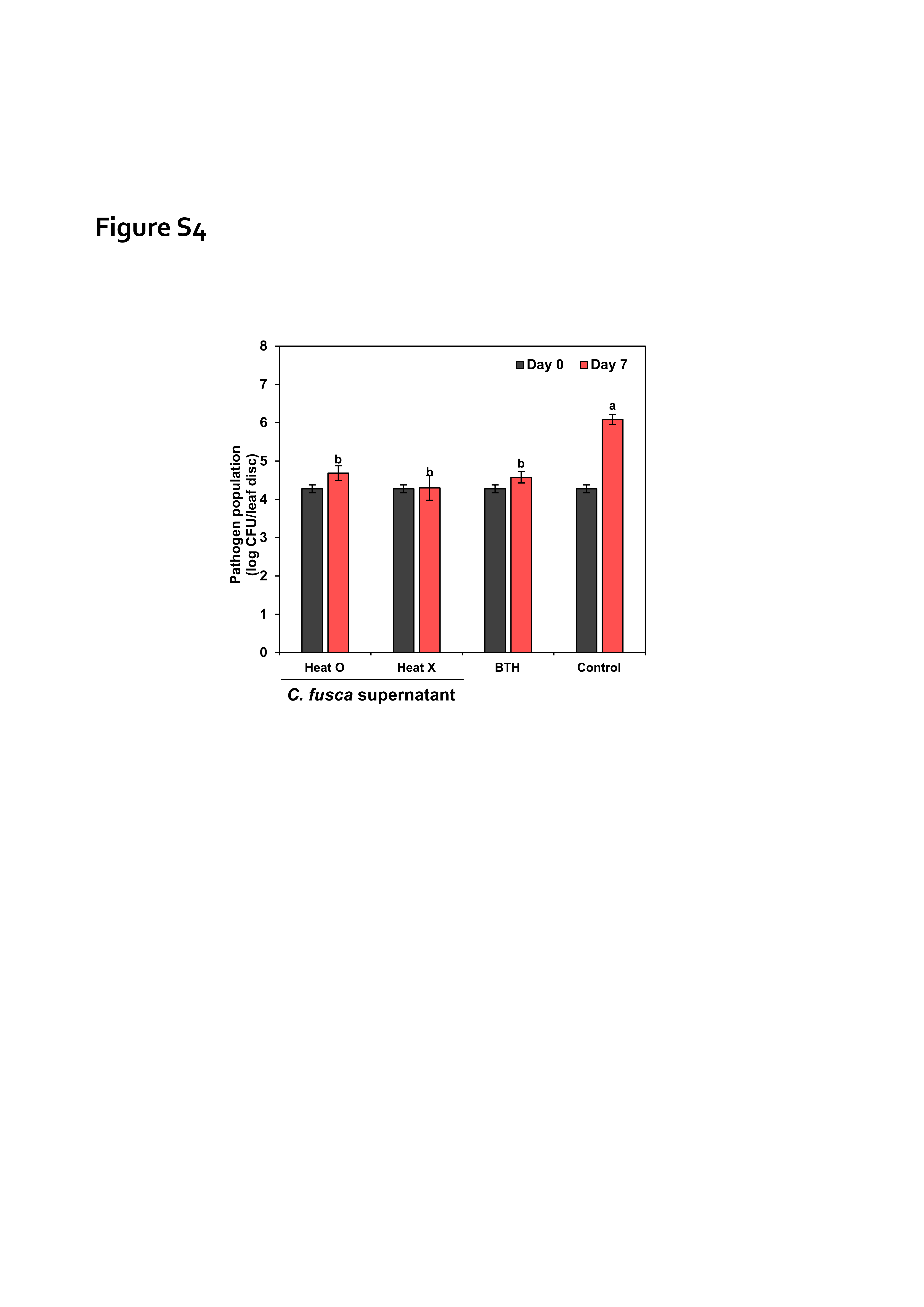

Supplement: Supplementary file 5 — Figure S4. Activation of C. fusca supernatant‐triggered induced resistance in Arabidopsis against Pto DC3000 with or without heat treatment. [file TPJ-102-761-s005.png]

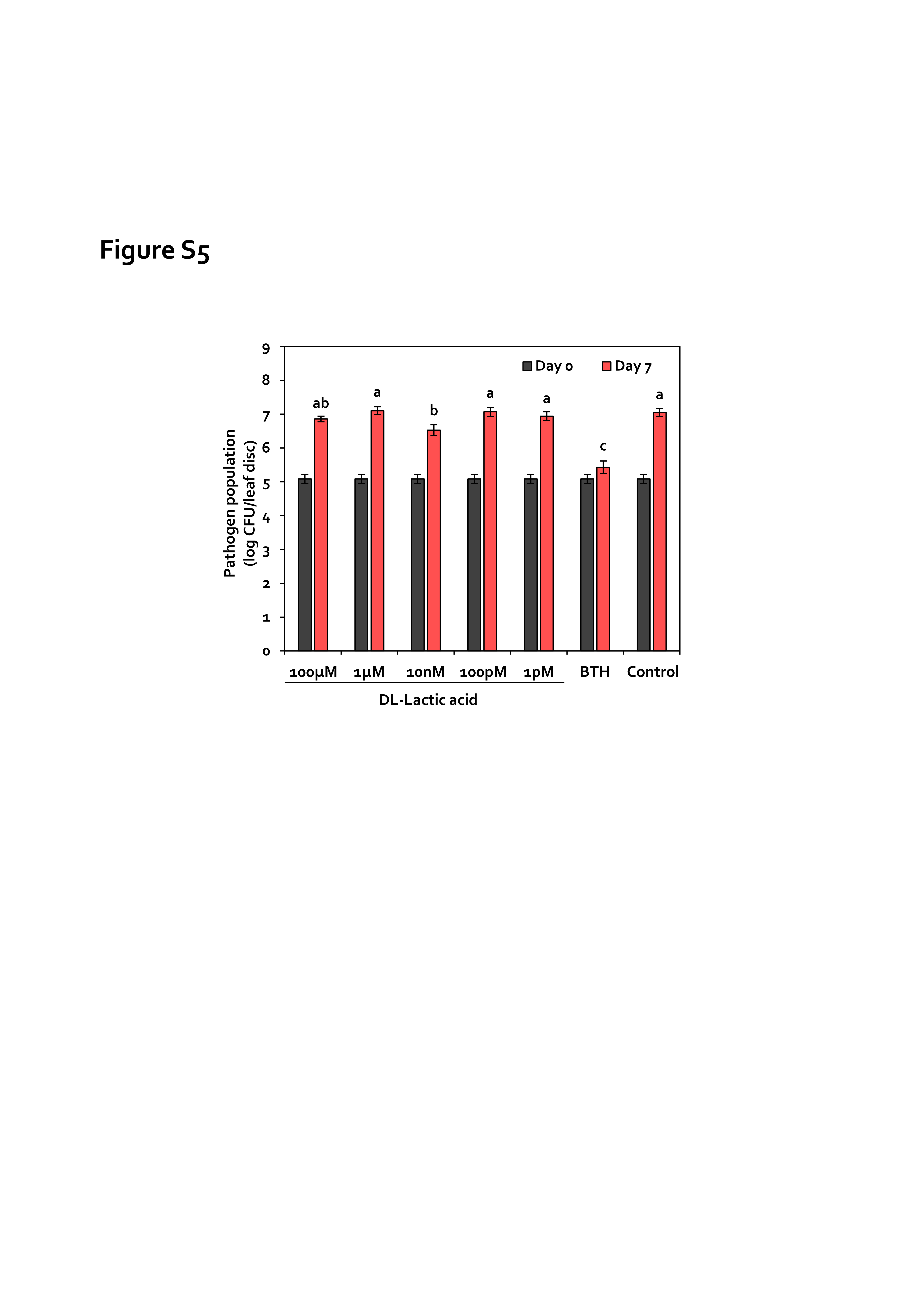

Supplement: Supplementary file 6 — Figure S5. Optimization of dl‐lactic acid concentration for eliciting induced resistance against Pto DC3000 in Arabidopsis. [file TPJ-102-761-s006.png]

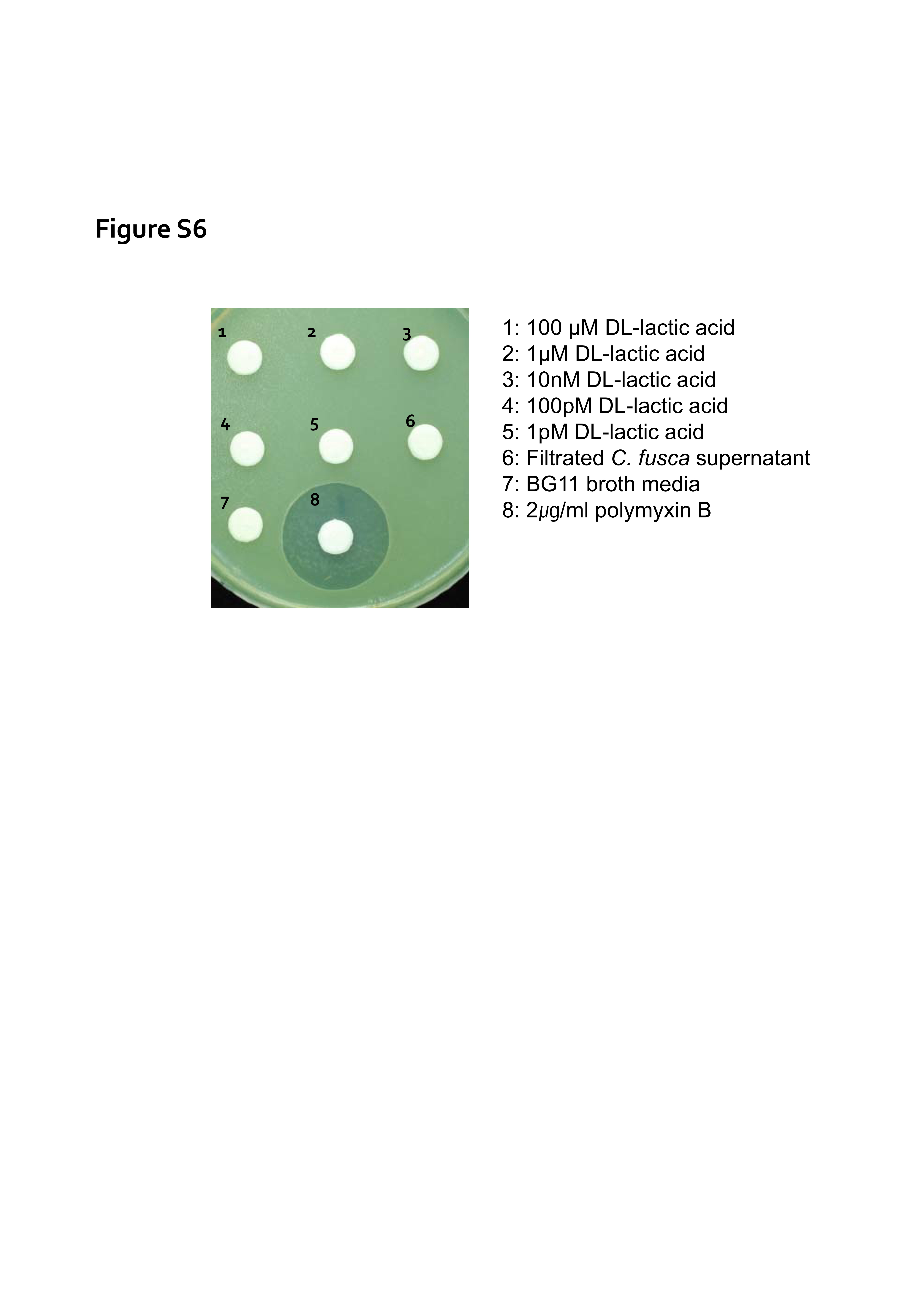

Supplement: Supplementary file 7 — Figure S6. Analysis of antagonism between filtered C. fusca supernatant or dl‐lactic acid and Pto DC3000. [file TPJ-102-761-s007.png]
