# Supplementary material for: d‐Lactic acid secreted by Chlorella fusca primes pattern‐triggered immunity against Pseudomonas syringae in Arabidopsis
Source: Plant J. 2020 Jan 27;102(4):761–78. doi: 10.1111/tpj.14661 (PMC7318130; doi:10.1111/tpj.14661)
Supplement: Supplementary file 8 [file TPJ-102-761-s008.docx]

**SUPPORTING INFORMATION**

**Table S1. List of primers used for qRT-PCR analysis.**

**Figure S1. Optimization of cell density and growth conditions of *Chlorella fusca* for biological control against *Pseudomonas syringae* pv. tomato DC3000 (*Pto* DC3000) in *Arabidopsis*.**

**Figure S2. Induced resistance-associated differentially expressed genes (DEGs) in *C. fusca*-treated *Arabidopsis* leaves at 0 and 12 hpi.**

**Figure S3. Gene ontology (GO) enrichment analysis of DEGs identified in *C. fusca*-treated *Arabidopsis* leaves at 12 hpi.**

**Figure S4.** **Activation of *C. fusca* supernatant-triggered induced resistance in *Arabidopsis* against *Pto* DC3000 with or without heat treatment.**

**Figure S5. Optimization of DL-lactic acid concentration for eliciting induced resistance against *Pto* DC3000 in *Arabidopsis*.**

**Figure S6. Analysis of antagonism between filtered *C. fusca* supernatant or DL-lactic acid and *Pto* DC3000.**
